# Essential Rule Derived from Thermodynamics and Kinetics Studies of Benzopyran Compounds

**DOI:** 10.3390/molecules28248039

**Published:** 2023-12-11

**Authors:** Baolong Chen, Xin Hu, Xiaoqing Zhu

**Affiliations:** 1 The State Key Laboratory of Elemento-Organic Chemistry, Collaborative Innovation Center of Chemical Science and Engineering, College of Chemistry, Nankai University, Tianjin 300071, China; 2 School of Chemistry and Chemical Engineering, Nanchang University, Nanchang 330031, China; huxin_huaxue@126.com

**Keywords:** benzopyran, thermodynamics, kinetics, essential rule

## Abstract

Compounds with benzopyran as the core structure play an important role in the total synthesis of antioxidants, drugs, and natural products. Herein, the thermodynamic data of benzopyran compounds and their intermediates were measured and calculated by combining thermodynamics with kinetics. The mechanism of reactions between four benzopyran compounds and organic hydride acceptors was proven to be a one-step hydride transfer. The thermodynamic properties of these compounds and their corresponding intermediates were elucidated. The rationality and accuracy of the electrochemical measurement method were proved. Furthermore, the essential rule of unique structures being present between the C–H bond and para-substituent constants on the benzene ring, as shown in previous studies, was investigated. A simultaneous correlation between thermodynamics and kinetics was found for the hydride transfer reaction, in which the reaction site is connected with the substituent through the benzene ring, a double bond, or a N atom. The likely reason for the correlation between thermodynamic and kinetic is that the benzene ring, double bond, or N atom have the role of transferring the electronic effect. This finding can be applied to the calculation of the activation energy of hydride self-exchange reactions, the prediction of kinetic isotope effects, and explorations of selective reduction processes of hydride transfer in such organic hydride compounds.

## 1. Introduction

**Scheme 1:**
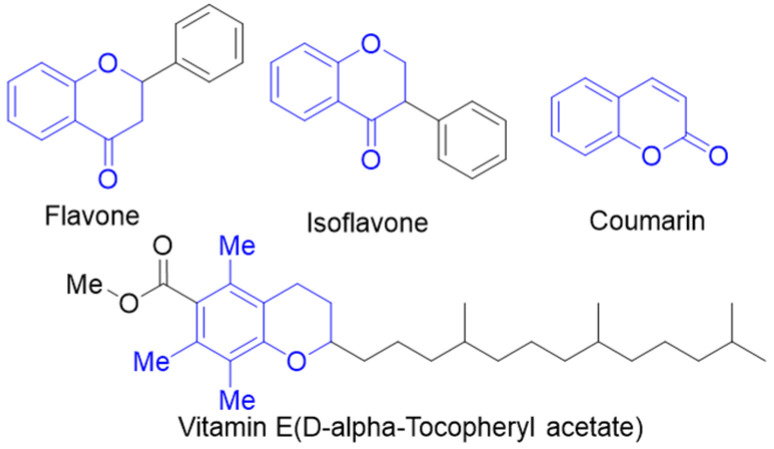
Structures of the benzopyran compounds.

Benzopyran is an important parent structure of natural products, such as flavone [1,2,3], isoflavonoid [4,5,6], coumarin [7,8,9], and vitamin E [10,11,12] (Scheme 1). One example that has received extensive research attention is anthocyanidin, a flavonoid compound, which is a water-soluble natural pigment found in plant petals that has various physiological functions, such as serving as an antioxidant, preventing cardiovascular and cerebrovascular diseases, protecting the liver, and inhibiting tumor-cell generation. The main cause of the physiological activity of anthocyanidin is that it often combines with various monosaccharides to form glucoside, which also exhibits a strong antioxidant effect on protecting vitamin A, thus reducing the oxidase activity and removing free radicals in the human body. Overall, benzopyran compounds play an important role as reducing agents in vivo. Numerous studies (Supporting Information) were conducted on the synthesis of such compounds, but quantitative studies on the C–H bond energy and the reaction activation energy of model compounds remain limited.

Considering the limited number of the aforementioned quantitative studies, as well as the thermodynamics and kinetic aspects of these compounds, herein, we determined the bond energy using the thermodynamic method, calculated the thermodynamic driving force of the reaction, investigated the reaction activity and selectivity using the kinetics method, and conducted a quantitative analysis of the chemical properties and hydride-transfer mechanism of the abovementioned benzopyran compounds and their intermediates. This study provides support for the data and a theoretical basis for the synthesis of these compounds and their roles in molecular biology.

**Scheme 2:**
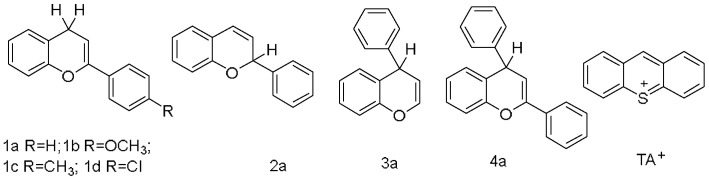
Structures of benzopyrane organic hydride donor compounds and the organic hydride acceptor compound TA**^+^**.

Scheme 2 shows the structures of the benzopyran hydride compounds synthesized in this study to investigate the reducing capacity. The oxygen atoms are located at the ortho and para positions in pyrane rings.

**Scheme 3:**
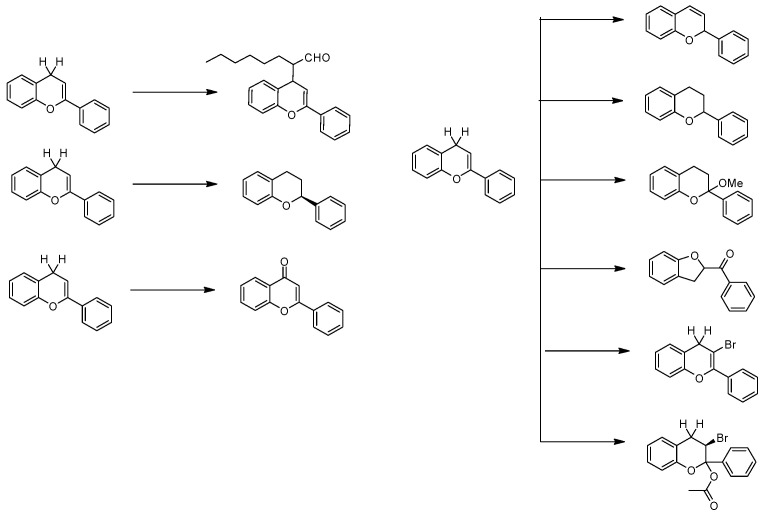
Current state of the organic synthesis of benzopyran compounds.

Over the years, **1a** has been loaded with aldehyde groups [13], achieved chiral reductions [14], formed the structure of chalcone [15], and transformed various other structures [16] (Scheme 3). The literature is relatively old, indicating that the research on the conversion of compounds has become stagnant. To the best of our knowledge, in the present study, we confirmed the direct formation of a pyranoid-ring positive ion using four hydride compounds, **1a**, **2a**, **3a** and **4a**, for the first time.

The transformation of compounds begins with the study of thermodynamics. The capacity of the benzopyrans to release hydride anions in vivo is referred to as hydride affinity, which represents the reducing capacity and directly affects the reactivity of these compounds with other compounds in vivo.

**Scheme 4:**
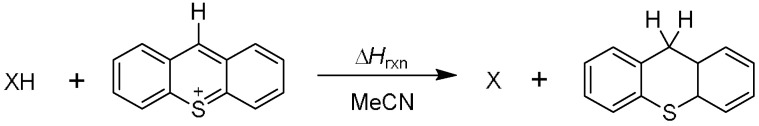
The representative hydride transfer reactions in this study.

Owing to the negligible interference in the ultraviolet range, acetonitrile is the most common solvent for obtaining molecular data in physical organic chemistry. The free hydride ion in it does not have a steady presence. The change in enthalpy for the process of benzopyran compounds accepting a hydride anion is exactly opposite to that in the heterolytic process of anions undergoing hydride transfer (Equation (1)). The thermodynamic driving force of hydride transfer depends on the capacity of the hydride donor to provide the hydride, and the capacity of the hydride acceptor to accept the hydride. X stands for hydride acceptor; similarly, XH stands for hydride donor. The hydride affinity of X in acetonitrile at 298 K ∆*H*_H−A_(X) can be obtained from the reaction enthalpy change in the corresponding hydride donor XH with a strong hydride acceptor, as shown in Equation (2). Scheme 4 depicts this reaction with the thiaxanthene cation.

∆*H*_H−A_(X) = −∆*H*_H−D_(XH) (1)

∆*H*_H−A_(X) = ∆*H* (TA^+^) − ∆*H*_rxn_ (2)

**Scheme 5:**
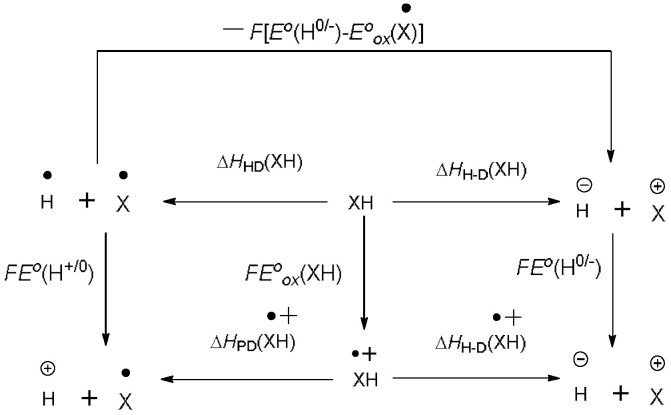
Three thermodynamic cycles were constructed based on the chemical process of XH releasing the hydride anion in acetonitrile.

A hydride anion comprises a proton and two electrons, providing three pathways for hydride transfer: (1) a one-step hydride anion transfer; (2) a hydrogen atom–electron transfer; (3) an electron-initiated multistep transfer, either e–H^+^–e or e–H^•^. The same pathways are available for the hydride anions of benzopyran hydride compounds. General hydride anion transfer mainly involves the bond energy changes described in the following paragraph (Scheme 5).

The hydride affinity ∆*H*_H−D_(XH) is defined as the molar enthalpy change for XH releasing a hydride anion in acetonitrile at 298 K. The hydrogen atom affinity ∆*H*_HD_(XH) is defined as the molar enthalpy change in XH releasing a hydrogen atom. For convenience, the notation ∆*H*_HD_(XH) is used instead of the more conventional notation ∆*H*_BDE_(XH). The hydrogen atom affinity ∆*H*_H−D_(XH^•+^) is defined as the molar enthalpy change in a radical cation X^•+^ releasing a hydride atom. The proton affinity ∆*H*_PD_ (XH^•+^) is defined as the molar enthalpy change in a radical cation X^•+^ releasing a proton.

The thermodynamic parameters of the reactants and reaction intermediates shown in Scheme 4 may be calculated from the electrochemical and calorimetric data; the corresponding derivation equations are shown in Equations (3)–(5). *E*^o^_red_(XH) is the reduction potential of benzopyran hydride, and *E*^o^red(X^•^) is the reduction potential of the intermediates of benzopyran hydride. We replaced ∆*H* with ∆*G*_ET_, referring to the literature value [17] to determine reversible potentials, considering *E*_1/2_(H^+/0^) = −2.307 (V vs. Fc^+/0^) and *E*_1/2_(H^0/−^) = −1.137 (V vs. Fc^+/0^) (Fc = ferrocene).

∆*H*_HD_(XH) = ∆*H*_H−D_(XH) − F [*E*^o^_ox_(X^•^) − *E*^o^(H^0/−^)] (3)

∆*H*_PD_(XH^•+^) = ∆*H*_HD_(XH) − F [*E*^o^_ox_(XH) − *E*^o^(H^+/0^)] (4)

∆*H*_HD_(XH•+) = ∆*H*_H−D_(XH) − F [*E*^o^_ox_(XH) − *E*^o^(H^0/−^)] (5)

## 2. Results

### 2.1. The Statement of Measurement Methods

**Figure 1:**
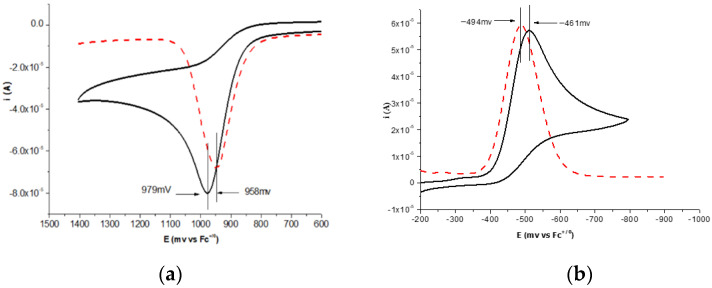
Cyclic voltammetry (CV) and Osteryoung square-wave voltammetry (OSWV) of (**a**) **2a** and (**b**) **2^+^** in deaerated acetonitrile containing 0.1 M n-Bu_4_NPF_6_ as the supporting electrolyte. Solid line: CV graph (sweep rate = 0.1 V/s); dashed line: OSWV graph.

Common electrochemical measurement methods mainly include cyclic voltammetry (CV) and Osteryoung square-wave voltammetry (OSWV). The detailed procedures for the methods used in our research are listed in the discussion section, and the reliability and accuracy of these methods are explained in detail. We used these methods to determine the single-electron reduction potentials of four types of benzopyran hydride compounds and their radical intermediates, as shown in Figure 1 and the Supporting Information. The obtained data, calculated based on data obtained using the OSWV method, are listed in Table 1.

**Figure 2:**
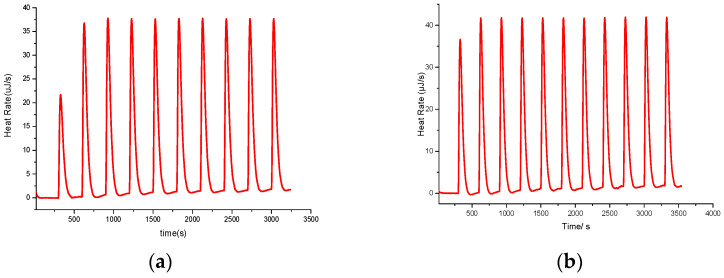
(**a**) Isothermal titration calorimetry (ITC) for the reaction heat of **1d** with TA^+^ClO_4_^−^ in acetonitrile at 298 K. Titration was conducted by adding 10-μL TA^+^ClO_4_^−^ (2.14 mM) every 300 s to acetonitrile containing **1d** (ca.10 mM). (**b**) ITC for the reaction heat of **2a** with TA^+^ClO_4_^−^ in acetonitrile at 298 K. Titration was conducted by adding 10-μL TA^+^ClO_4_^−^ (1.5 mM) every 300 s into acetonitrile containing **2a** (ca. 10 mM).

In this study, ∆*H*_rxn_ is the molar enthalpy change in the reaction in acetonitrile at 298 K, as calculated in Equation (2) using data obtained from titration calorimetry. We first determined the ∆*H*_rxn_ of the reaction between TA^+^ClO_4_^−^ and a suitable hydride donor using isothermal titration calorimetry (ITC). In our previous study [18], we calibrated the hydride affinity of TA^+^ClO_4_^−^, ∆*H*(TA^+^), as −100.5 kcal/mol. Similarly, we determined the ∆*H*_rxn_ of the reaction between XH and TA^+^ClO_4_^−^ (Figure 2 and the Supporting Information). Relevant data are listed in Table 1.

**Figure 3:**
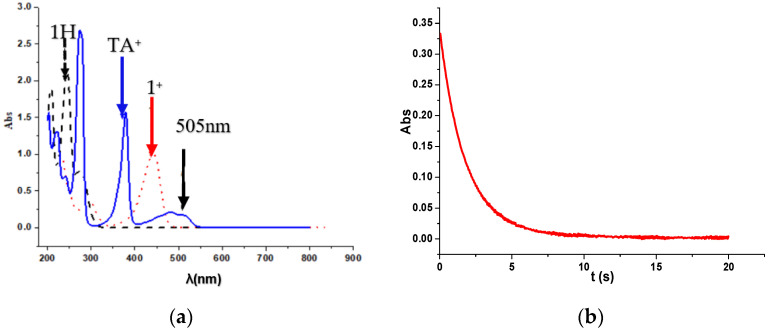
(**a**) Absorbance spectra of the reactants and corresponding products in acetonitrile at 298 K. The concentration of all compounds is 0.1 mM/L. The reaction stoichiometry of **1a**: TA^+^ is 1:1. (**b**) Profile of the UV-vis absorbance of TA^+^ at λ_max_ = 505 nm during the hydride transfer from **1a** (R = H) to TA^+^. Conditions: 0.1 mM/L TA^+^ and 2.0 mM/L **1a** in acetonitrile at 298 K.

Since the ultraviolet curves of **1a** and the hydride acceptor do not completely overlap (Figure 3a), the apparent rate constant *k_obs_* of this reaction can be determined (Figure 3b). The kinetic experiment was carried out by UV-vis stopped-flow spectrophotometry under pseudo-first-order conditions with an excess of **1a** over TA^+^ in acetonitrile at 298 K (Supporting Information).

### 2.2. Data Collection Method

Using the electrochemical data *E*_1/2_(H^+/0^), *E*_1/2_(H^0/−^), and Δ*H*(TA^+^), we substituted the thermodynamic data (directly measured) into Equations (3)–(5) to obtain the hydride affinity Δ*H*_H−D_(XH) and hydrogen atom affinity Δ*H*_HD_(XH) of benzopyran hydride compounds, as well as the hydrogen atom affinity Δ*H*_HD_(XH^•+^) and proton affinity Δ*H*_PD_(XH^•+^) of the corresponding radical anions.

Accordingly, the second-order rate constant *k*_2_ is equal to the apparent rate constant *k*_obs_ divided by the concentration of the hydride donor. All data are summarized in Table 2.

### 2.3. Data Analysis Process

**Scheme 6:**
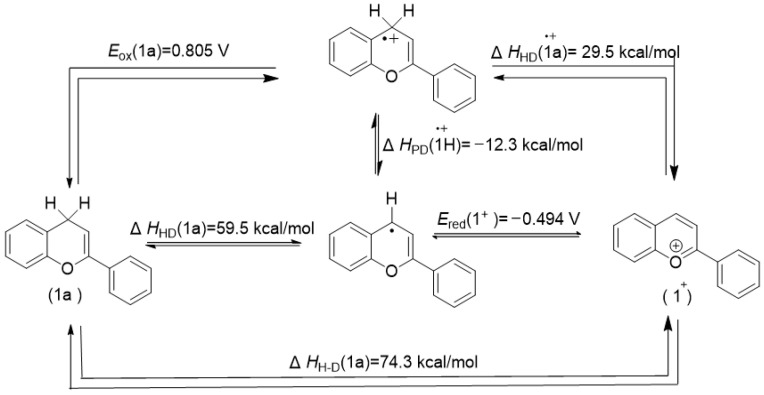
Thermodynamic characteristics diagram of **1a**.

According to the mentioned electrochemical data and isothermal titration calorimetry (ITC) data, the molecular identity card (Molecular ID) containing the thermodynamic parameters of **1a** is depicted in Scheme 6. Similar diagrams are shown for **2a**, **3a**, and **4a** in the Supporting Information. Table 3 presents a summary of the thermodynamics data and properties of **1a** compounds and their intermediates.

**Scheme 7:**
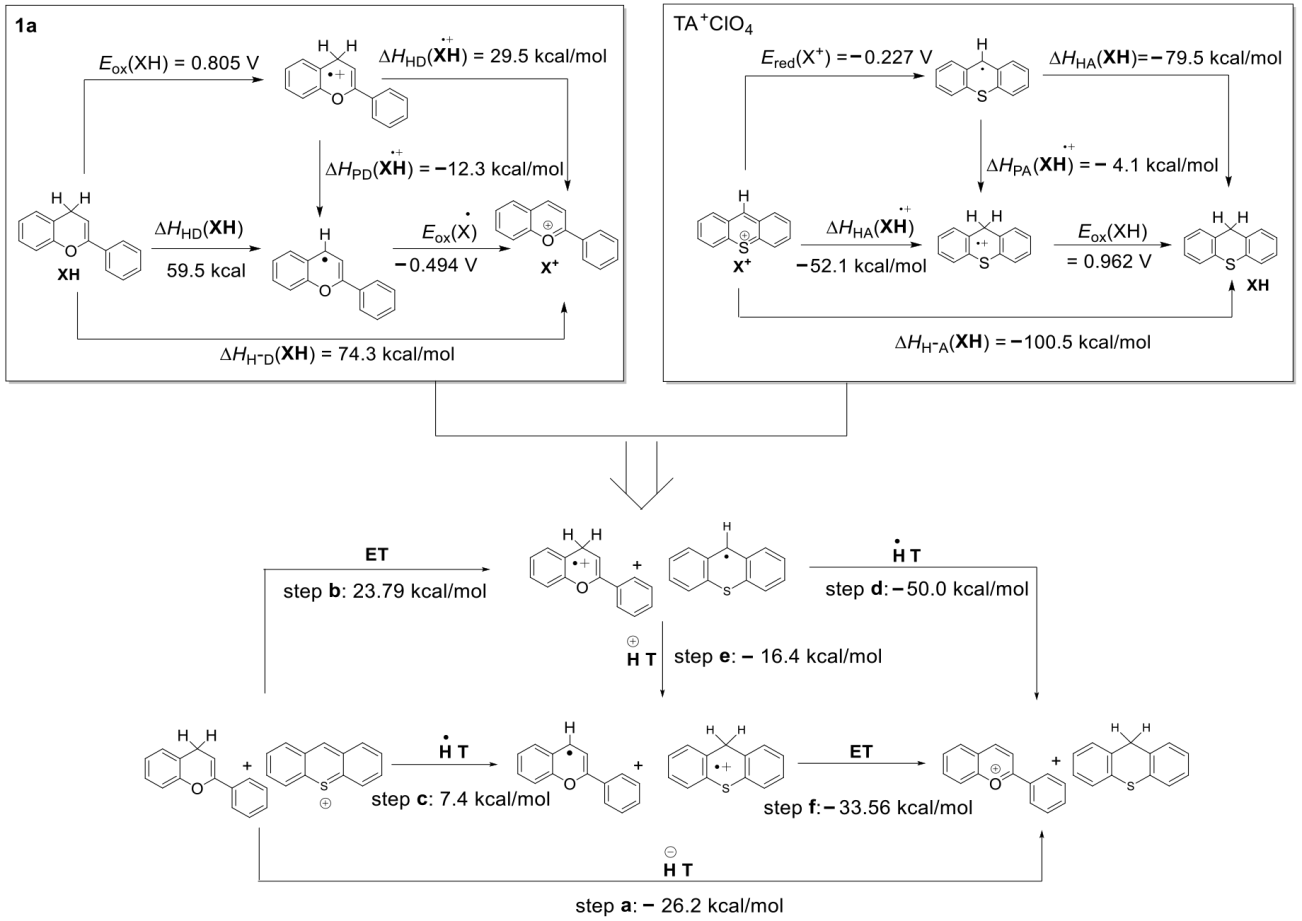
Thermodynamic energy values (in units of kcal/mol) of the steps of possible hydride transfer mechanisms between **1a** (R = H) and TA^+^ClO_4_^−^ in acetonitrile at 298 K.

From the perspective of thermodynamics, the molecular identities of **1a** and TA^+^ClO_4_^−^ can be combined to obtain the thermodynamic platform of the reaction (Scheme 7). The enthalpy change in the thermodynamic platform represents the addition of the corresponding enthalpy change in the Molecular ID. The Gibbs free energy of step a (one-step hydride transfer) is −26.2 kcal/mol, which is considerably smaller than the Gibbs free energies of step b and step c, suggesting that the reaction is a one-step hydride transfer.

**Figure 4:**
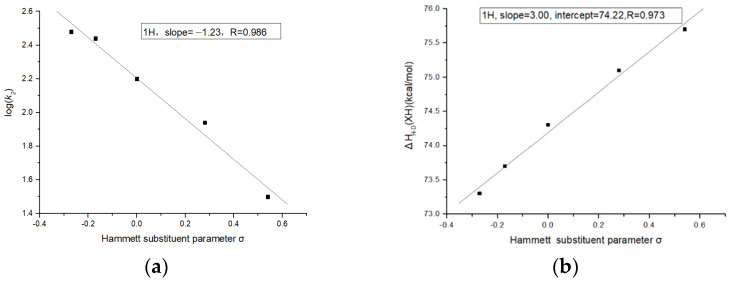
Linear correlation curves of (**a**) log *k*_2_ and (**b**) Δ*H*_H-__D_(XH) with the Hammett substituent parameter σ for **1a** in acetonitrile at 298 K.

From the perspective of kinetics, the logarithm of the second-order rate constant of the reaction of **1a** with TA^+^ClO_4_^−^, log *k_2_*, and therefore the activation energy, show a strong linear dependence on the benzene para substituent constant σ_p_, as shown by the plot in Figure 4a. The slope of this plot, i.e., the reaction rate constant ρ (ρ_1_ = −1.23), is negative, indicating an accumulation of positive charge on the pyran ring at the reaction center in the transition state of the reaction. Therefore, the reaction is more likely to occur via a one-step hydride transfer or electron transfer mechanism.

**Scheme 8:**
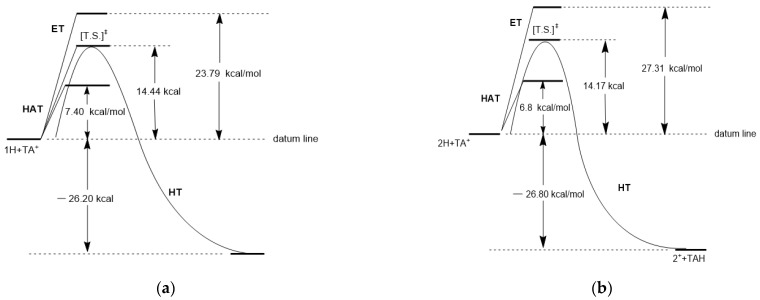
Comparison of energy state changes for the three possible initial steps of hydride transfer and activation free energy of the hydride transfer from (**a**) **1a** (G = H) and (**b**) **2a** to TA^+^. The symbol “‡” stands for activation energy.

Analysis of the thermodynamic data of benzopyran hydride compounds shows that the hydride affinity Δ*H*_HD_(XH) for **1a** exhibits an excellent linear dependence on the benzene ring para**-**substituent constant σ_p_ (Figure 4b). The thermokinetics correlation of hydride compounds shown in Figure 4a,b has been investigated many times in our previous studies. Representative structures of compounds that follow this correlation are illustrated in Scheme 8. The ordinate and abscissa indicate the change in the energy and reaction progress, respectively, and the curve represents the hydride transfer reaction. The values of **Δ**G^≠^ are derived from kinetic measurements and are attributed to the initial hydride transfer. Because of the extensive validity of this relation between the substituent constant of the benzene ring para position and the reaction site in the hydride transfer reactions, we predict that such a thermokinetics correlation will also occur for molecules **2a**, **3a**, and **4a**.

## 3. Discussion

### 3.1. The Statement of Novel Findings

Over the years, our research group has explored many interesting phenomena of organic hydride compounds.

In Zhang Min-Tian’s study [19], a five-membered heterocyclic ring was used to prove the linearity of the relation between the substituent constants of the benzene ring near the reaction site of benzimidazole and the hydride affinity of the reacting C–H bond. The ortho, para, meta, or coexisting para/meta benzene ring substituent constants of molecules 3H and 4H, the benzene ring substituent constants at the distal reaction site on benzimidazole 2H, the meta or para benzene ring substituent constants at the reaction site on benzothiazole 6H, and the para substituent constants close to the reaction site on benzoxazole 7H were all found to have a linear relation between the hydride affinity of the C–H bond and the reaction site.

We next investigated the reaction of acridine salts with these five-membered heterocyclic ring compounds. Molecular identification, thermodynamic analyses, and kinetics studies all proved that the reaction occurred via hydride transfer. Therefore, the thermokinetics correlation phenomenon of hydride compounds was repeatedly confirmed.

In Yang Jin-Dong’s study [20], which focused on dihydropyridine, the relationship between the hydride transfer activation energy at site 2 and site 4 of numerous dihydropyridine derivatives and the substituent constant was investigated via kinetics studies, proving that the relation between them is also linear.

In this study, which focused on the pyran ring, a combination of kinetics and thermodynamics proved the linear dependence of the hydride affinity of the activation site on the benzopyran ring on the substituent constants. The hydride transfer activation energy showed a similar linear dependence on the substituent constants. Therefore, a simultaneous correlation of thermodynamics and kinetics exists between these factors.

**Scheme 9:**
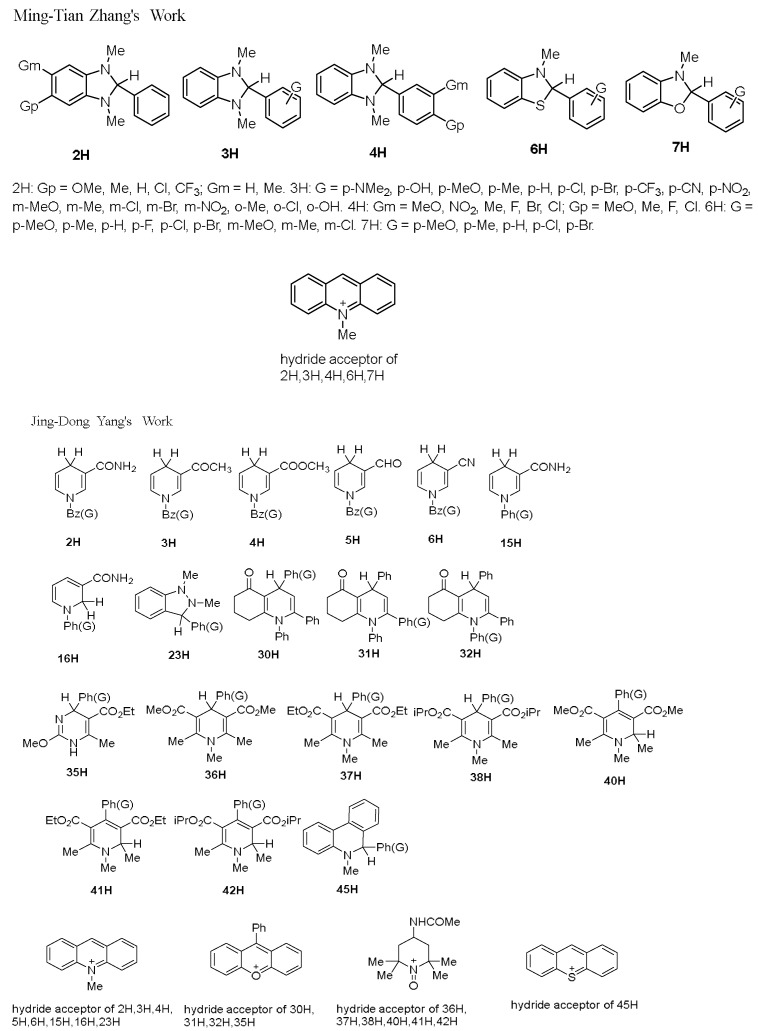
Synthesized compounds applied to kinetics and thermodynamics research in previous work from our laboratory [19,20].

Through careful study of the compounds in Scheme 9 and this research on the new compound structure reported herein, a simultaneous correlation between thermodynamics and kinetics was found for the hydride transfer reaction, in which the reaction site is connected with the substituent through the benzene ring, a double bond, or a N atom. This correlation is most likely related to the hybridization of the delocalized π bonds of the benzene ring and the σ component of the double bond. The possibility of multiple valence states for the N atom (−3, 0, +1, +2, +3, +4, and +5) confers the ability to store and release electrons. These three structures (π bonds, σ component, N atom) have a better capacity to store and release electrons and better thermodynamics and kinetics factors than other simple chemical bonds for hydride transfer in the molecules.

### 3.2. Accuracy and Reliability of the Novel Findings

Our research team identified the correct method for the electrochemical measurement of organic chemical potentials in solution many years ago. This proved the rationality and accuracy of this method and ensured the authenticity and reliability of the above thermodynamic data.

In fact, scaling and measuring the standard electrode potential of organic compounds to analyze the properties of such compounds are challenging tasks. Because organic compounds are usually insoluble in water but easily dissolve in organic media, some traditional methods that are commonly used in the laboratory to determine the standard electrode potential of inorganic ions in aqueous media are not suitable for the determination of the same for organic compounds. To solve this problem, usually, two methods are employed to scale the standard electrode potential of organic compounds, viz. cyclic voltammetry and square wave voltammetry, as reported in previous studies.

The principle of cyclic voltammetry is that the oxidation state of a tested organic compound is reduced on the cathode, and the oxidation of the reduced state of the tested compound on the anode generates electronic flow. If both the oxidation and reduction states of the tested organic compound can exist stably on the electrode surface, then reversible double peaks with ~60 mV distance appear on the cyclic voltammogram. As the two peaks are both asymmetric, the positions of the reduction current peak (*E*_red_) and oxidation current peak (*E*_ox_) change as the concentration of the tested organic compound (i.e., the current intensity) changes. However, their average value, that is, the half-wave potential *E*_1/2_ = 1/2 (*E*_red_ + *E*_ox_), will not change. Therefore, chemists usually use the half-wave potential as the quantitative scale. However, if one of the oxidation or reduction states of the organic compound is unstable during the electrode reaction, that is, a subsequent reaction occurs, then the concentrations are not equal to each other in the reversible change process, and the reversible double peaks are not shown to have equal intensities; in this case, only a single peak is observed. As the redox current peak position of the organic compound is dependent on the nature of the organic compound as well as its concentration, it is impossible to determine the so-called half-wave potential.

However, the results of several reported experiments show that, for most organic compounds, if their concentration is very low, for example, less than 5 × 10^−5^ M, then the difference between the position of the irreversible current peak and its corresponding half-wave potential *E*_1/2_ is generally ~30 mV.

To overcome the defects in cyclic voltammetry, Osteryong et al. established square wave voltammetry. Its basic principle is similar to that of cyclic voltammetry, that is, the standard electrode potential of the organic compound is scaled by the position of the reduction current peak (*E*_red_) and the position of the oxidation current peak (*E*_ox_) of the tested organic compound. However, two differences are observed: (i) The positions of the reduction current peak (*E*_red_) and oxidation current peak (*E*_ox_) in a square wave voltammogram are equal, which implies that *E*_red_ and *E*_ox_ can be directly used to scale the electrode potential of the examined organic compound. (ii) The left and right sides of the current peaks are nearly symmetrical, which implies that *E*_red_ and *E*_ox_ extracted from the square wave voltammogram remain almost unchanged when the concentrations of the oxidation and reduction states of the examined compounds change because the peak positions remain unchanged during irreversible electrochemical processes. This result was validated through experiments in our previous study [19].

If the current peak in the square wave voltammogram is significantly asymmetric from left to right due to the subsequent reaction [21], then the position of the current peak in the square wave voltammogram will significantly change due to the subsequent reaction. At this time, the position of the current peak in the square wave voltammogram cannot be used as the standard electrode potential of the examined compound. However, this paper [17] also indicates that if the concentration of the measured substance is very low, then the position of current peak in the square wave voltammogramis can still reliably scale the standard electrode potential of the examined substance; this is because, at low concentrations, the current intensity (i.e., the height of the current peak) in the square wave voltammogram is low, and the subsequent reaction only negligibly influences the position of the current peak. In our case, the square wave voltammogram shows symmetric (or almost symmetric) current peaks for all the organic compounds; therefore, the results of the square wave voltammetry experiments reported in this paper are reliable.

The acetonitrile was strictly cleaned of water and oxides. Silver nitrate and Fc content, and the purity of the compound, were high. We repeated the determination of the potential of the compound in Zhang’s work several months later, and found that the difference was almost zero. In addition, after determining the potential 3–5 times, we replaced the silver nitrate solution and remeasured the standard potential of Fc. This was our verification and calibration process.

### 3.3. The Application Value of Novel Findings

First, the findings reported in this paper provide data that support the development of physical organic chemistry.

**Scheme 10:**
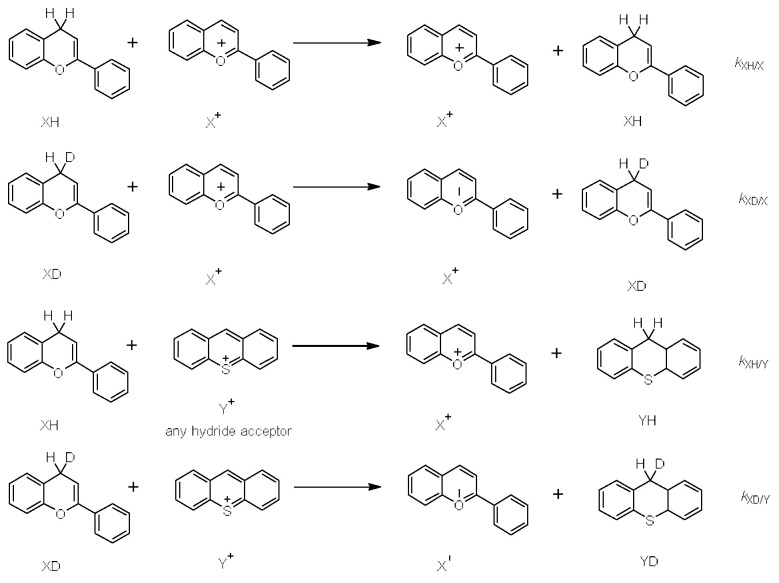
Representative hydride reactions.

Based on the Eyring equation: *k*_2_ = (k_B_T/h)exp(−**Δ**G^≠^/RT), the kinetic data in this paper can supplement the data of this compound: the activation energies of hydride self-exchange reactions (ΔG^≠^_XH/X_; XH + X^+^ → X^+^ + XH) [20], and KIEself values of hydride self-exchange reactions (KIE_XH/X_ = *k*_XH/X_/*k*_XD/X_) [22] (Scheme 10). These two kinetic quantities are the essential kinetic properties of hydride compounds and are of great significance. According to Zhu’s kinetic model [22], (KIE_XH/Y_)^2^ = KIE_XH/X_ × KIE_YH/Y_, our research group has successfully predicted thousands of hydride isotope effects. Therefore, this model can also be used to predict the kinetic isotope effects of the cross-hydride reactions of benzopyran compounds. Because of the presence of two hydrogen atoms at the reaction site in hydride compound **1a**, the kinetic isotope effect cannot be observed during experiments: therefore, kinetic predictions are crucial for determining the isotope effect. We called this kinetic significance.

Second, the findings reported in this paper provide support for organic synthetic chemistry.

**Scheme 11:**
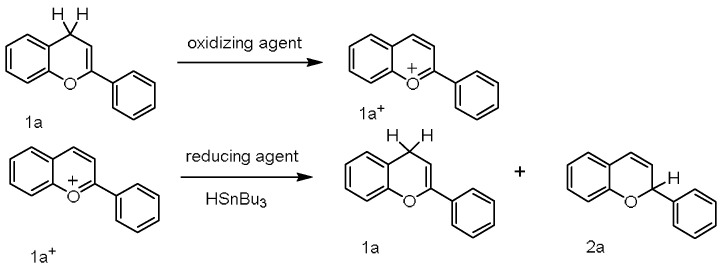
The oxidation and reduction of benzopyran compounds.

There is no steric hindrance at the **1a** activation site, and the hydride reaction is only affected by the abstracting hydride capacity of the oxidizing agent. When a hydride compound **1a** is oxidized to **1a^+^**, what is an oxidizing agent’s minimum capacity to abstract hydride? In addition, compound **1a^+^** (R = H) is reduced to form **1a**. There is only one report on the application of HSnBu_3_ [23] (Scheme 11). HSnBu_3_ is a dangerous compound, just like n-Butyllithium, and −40 °C is a harsh reaction condition. Therefore, the selective reduction of this compound is almost non-existent. Does a selective reduction depend on the capacity of the reducing agent to the donor hydride? Or is the hydride reaction itself selective? These problems need to be supported by the thermodynamic data in this paper, combined with the results of future **1a^+^** reduction experiments, to draw definite conclusions and promote the development of organic synthesis research. We called this thermodynamic significance.

### 3.4. Assessment of Novel Findings

Our findings are compared and evaluated here.

Compared with conventional organic synthesis, our study demonstrates a novel one-step hydride reaction for the generation of pyranoid-ring positive ions. Organic synthesis research is promoted to the stage of quantitative analysis. Our study also provides data supporting the theoretical research on physical organic chemistry. In the study of density functionals, for example, the internet Bond-Energy Databank was created by the Department of Chemistry of Tsinghua University to collect a large amount of thermodynamic data, and Yang Li also used the density functional method to calculate the kinetic data of hydride compounds [24]. These studies are characterized by rich contents, convenience, and continuous updates. In comparison, our research still requires the synthesis of compounds, data are scarce, and the required time is slightly longer. However, the data are much more realistic and reliable.

## 4. Experimental Procedures

### 4.1. Measurment of Redox Potentials

The electrochemical experiments were carried out by cyclic voltammetry (CV) and osteryoung square wave voltammetry (OSWV) using BAS100B electrochemical apparatus in deaered acetonitrile under argon atmosphere at 298 K, as described previously. nBu_4_NPF_6_ (0.1 M) in acetonitrile was employed as the supporting electrolyte. A standard three-electrode cell consists of a glassy carbon disk as a work electrode, a platinum wire as a counter-electrode, and 0.1 M AgNO_3_/Ag (in 0.1 M n-Bu_4_NPF^6−^ acetonitrile) as the reference electrode. The ferrocenium/ferrocene redox couple (Fc^+/0^) was taken as the internal standard. The reproducibilities of the potentials were usually ≤5 mV for ionic species and ≤10 mV for neutral species.

**Figure 5:**
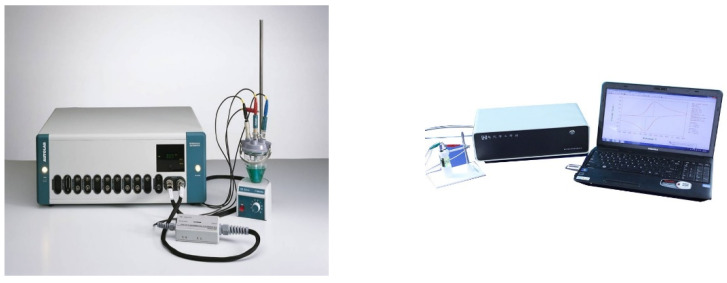
Electrochemical apparatus and its display screen.

**Figure 6:**
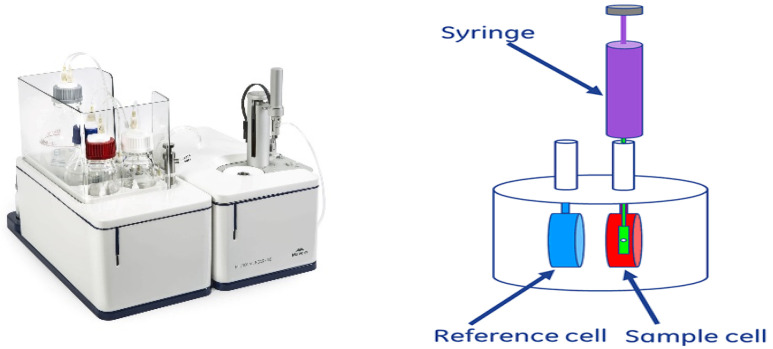
Isothermal titration calorimeter and sample injection.

**Figure 7:**
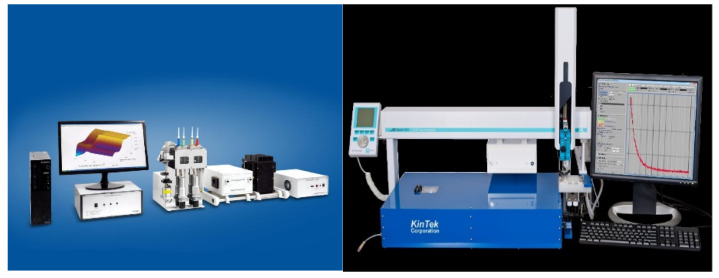
Stopped-flow spectrophotometer and its display screen.

Since the equipment in our group was too old, we present the latest applicable instruments to the readers in Figure 5, Figure 6 and Figure 7.

### 4.2. Isothermal Titration Calorimetry (ITC)

The titration experiments were performed on a CSC4200 isothermal titration calorimeter (Computer Sciences Corporation, Tysons, VG, USA) in acetonitrile at 298 K, as described previously. The performance of the calorimeter was checked by measuring the standard heat of the neutralization of an aqueous solution of sodium hydroxide with a standard aqueous HCl solution. The solvents used in the experiment were anhydrous and anaerobic acetonitrile. TA^+^ClO_4_^−^ (1 mM) was used as the titration solution; 1 mL carbonyl compound anion XH was used as the reaction solution. The experiment was determined at 298 K, the balance time was 400 s, and the titration time was 400 s. The reaction heat was obtained by integrating the area of each peak (except the first peak) in the titration curve. The test was repeated at least 5 times for each sample, and the reaction heat error was ≤±0.5 kcal/mol. Note: typically, the first injection contains less heat than expected. This is often due to diffusion across the tip of the needle or difficulties in positioning the buret drive.

### 4.3. Kinetics Measurment and Calculation Process

The kinetic runs were performed on an Applied Photophysics SX.18MV-R stopped-flow spectrophotometer at 298 K in acetonitrile. The SX.18MV-R, connected to a superthermostat circulating bath to regulate the temperature of cell compartments, has a dead time of about 1 ms with very high sensitivity. All solutions used for kinetics were prepared in an Ar-filled glovebox. These measurements of the reactions were under pseudo-first-order conditions with excess hydride donor concentrations at least 15 times larger than those of the hydride acceptor. The progress of the reaction was monitored by following the decrease in TA^+^ absorption at 505 nm in the visible region. All runs were repeated more than five times to ensure the reliability of the data.

*k*_2_ (M^−1^ s^−1^) is the second rate constant of the hydride transfer in acetonitrile at 298 K, derived from experimental measurements using a stoped-flow UV-vis spectrometer, and the uncertainty is smaller than 5%. Usually, the concentration of the hydride acceptor is 0.1 mmol/L; the concentration of hydride donor is 2.0 mmol/L.

According to Eyring equation:

*k*_2_ = (k_B_T/h)exp(−**Δ**G^≠^/RT)

when T = 298 K, we substitute the constants into Equation (1) to obtain the relationship between the activation free energy **Δ**G^≠^ and the second-order rate constant *k*_2_.

**Δ**G^≠^ = 1.36373 × (12.7926 − lg *k*_2_)

## 5. Conclusions

First, this study contributes to physical organic chemistry research.

A small database was established as follows:

1. According to the analysis of kinetics and thermodynamics data for the reactions between four types of benzofuran compounds with TA^+^ClO_4_^−^, along with the Molecular ID and reaction thermodynamic platforms, were obtained, proving that these reactions follow a one-step hydride transfer mechanism.
2. For the hydride transfer reaction, the hydride affinity of the C–H bond at the reaction site and the activation energy of the reaction exhibited excellent linear dependence on the benzene ring para substituent constants. A simultaneous correlation between thermodynamics and kinetics was predicted to appear in the hydride transfer reactions of the **2a**, **3a**, and **4a** compounds.

This original database was expanded. The simultaneous correlation between the thermodynamics and kinetics observed in studies of numerous reactions was summarized. Because the benzene ring, double bond, and N atom all performed the function of transferring the substituent effect (including thermodynamics and kinetics effects), the hydride affinity in hundreds of structures derived from a benzoheterocyclic parent or dihydropyridine parent, and the reaction activation energy displayed an excellent linear dependence on the substituent constants.

The findings reported in this paper provide data supporing the development of physical organic chemistry and lay the foundation for the organic synthesis of benzopyran compounds. The proposed method can be applied to determine the reaction mechanisms of compounds with other structures in the future.

Second, this study contributes to research in analytical chemistry.

In this paper, the reliability and accuracy of the method used to measure the potential of organic compounds in a solution are described in detail.

Third, the presented results could help to improve the understanding of beginners studying organic chemistry.

This study can provide beginners with an overview of the thermodynamic and kinetic study of organic compounds.

## Figures and Tables

**Table 1:** Reaction enthalpy changes of **1**, **2a**, **3a**, and **4a** with TA^+^ClO_4_^−^ and the redox potentials of relative species in acetonitrile at 298 K, which were measured via CV and OSWV, respectively.

Hydride (XH)	Δ*H*_rxn_^a^	*E*_ox_ (XH) ^b^		*E*_ox_(X^•^) ^b^	
		CV	OSWV	CV	OSWV
**1b**	27.2	0.711	0.680	−0.556	−0.584
**1c**	26.8	0.754	0.725	−0.502	−0.530
**1a**	26.2	0.835	0.805	−0.461	−0.494
**1d**	25.4	0.902	0.870	−0.412	−0.440
**2a**	26.8	0.979	0.958	−0.461	−0.494
**3a**	9.8	0.940	0.925	−0.505	−0.528
**4a**	14.3	1.012	0.997	−0.549	−0.570
TA^+^ClO_4_^−^		0.990	0.962	−0.238	−0.227

^a^ The values of Δ*H*_rxn_ were obtained from the reaction heats calculated using Equation (2) by switching the sign; they were measured via titration calorimetry in acetonitrile at 298 K. The data, presented in kcal/mol, were the average values of at least three independent runs. The reproducibility was ≤0.5 kcal/mol. ^b^ The values of Δ*E*_ox_ were measured using CV and OSWV methods in acetonitrile at 298 K, in units of volts vs. Fc^0/+^ and reproducible to 5 mV or better.

**Table 2:** Enthalpy changes in XH to release a hydride anion and to release a neutral hydrogen atom, enthalpy changes in XH**^•+^** to release a proton and to release a neutral hydrogen atom(kcal·mol^−1^), and second-order rate constants in acetonitrile at 298 K.

XH	Δ*H*_H_-_D_(XH) ^a^	Δ*H*_HD_(XH) ^b^	Δ*H*_PD_(XH^•+^) ^b^	Δ*H*_HD_(XH^•+^) ^b^	*k*_2_ (M^−1^S^−1^) ^c^
**1b**	73.3	60.6	−8.3	31.4	306.52
**1c**	73.7	59.7	−10.2	30.8	290.83
**1a**	74.3	59.5	−12.3	29.5	161.93
**1d**	75.1	59.0	−14.2	28.8	90.44
**2a**	83.7	58.9	−16.4	25.4	72.26
**3a**	90.7	76.7	−2.2	43.2	2.76
**4a**	86.2	73.1	−3.0	37.0	11.73

^a^ The Δ*H*_H−D_ (XH) values of **1, 2a, 3a,** and **4a** were estimated from Equation (2). ^b^ Δ*H*_HD_(XH), Δ*H*_PD_(XH**^+^**^•^), and Δ*H*_HD_(XH**^+^**^•^) were estimated using Equations (3)–(5), respectively. We selected the redox potentials of XH and X^+^, measured via OSWV, as *E*_ox_(XH) and *E*_ox_(X^•^), respectively (as recorded in Table 1), because the values obtained via OSWV were closer to the corresponding standard redox potentials than the values obtained via CV in this study. ^c^ *k*_2_ is obtained from experimental measurements by a stopped-flow method. The uncertainty is smaller than 5%.

**Table 3:** Chemical characteristic properties of the members of the **1a** family according to the thermodynamic characteristics diagram of **1a**.

Species	Thermodynamic Parameters	Diagnoses of the Characteristic Properties
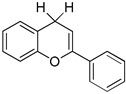	Δ*H*_H_-_D_(**1a**) = 74.3 kcal/mol Δ*H*_HD_(**1a**) = 59.5 kcal/mol *E*_ox_(**1a**) = 0.805 V	moderately strong hydride donor strong hydrogen donor and antioxidant weak one-e^−^ reductant
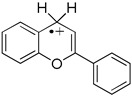	Δ*H*_PD_(**1a^•+^**) = −12.3kcal/mol Δ*H*_HD_(**1a^•+^**) = 29.5 kcal/mol *E*_red_(**1a^•+^**) = 0.805 V	very strong organic acid good hydrogen donor and antioxidant strong one-e^−^ oxidant
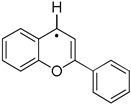	Δ*H*_PA_(**1a^•^**) = 12.3 kcal/mol Δ*H*_HA_(a**1^•^**) = −59.5 kcal/mol *E*_ox_(**1a^•^**) = −0.494V	weak base mildly strong hydrogen acceptor strong one-e^−^ reductant
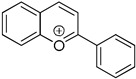	Δ*H*_H_-_A_(**1a^+^**) = −74.3 kcal/mol Δ*H*_HA_(**1a^+^**) = −29.5 kcal/mol *E*_red_(**1a^+^**) = −0.494 V	strong hydride acceptor strong hydrogen acceptor weak one-e^−^ oxidant

## Data Availability

Data are contained within the article and supplementary materials.

## Supplementary Materials

The following supporting information can be downloaded at: https://www.mdpi.com/article/10.3390/molecules28248039/s1, Figure S1: The route of all organic compounds synthesis. Figure S2: Decay of the 505 nm absorbance of TA^+^ClO_4_^−^ (1.0 mM) following the addition of 1b (20 mM) in deaerated anhydrous acetonitrile at 298 K (black line) and the fit (red line) using pseudo-first-order kinetic model. *k*_2_ = 306.52 M^−1^S^−1^. Figure S3: Decay of the 505 nm absorbance of TA^+^ClO_4_^−^ (1.0 mM) following the addition of 1d (20 mM) in deaerated anhydrous acetonitrile at 298 K (black line) and the fit (red line) using pseudo-first-order kinetic model. *k*_2_ = 90.44 M^−1^S^−1^. Figure S4: Decay of the 505 nm absorbance of TA^+^ClO_4_^−^ (1.0 mM) following the addition of 1a (20 mM) in deaerated anhydrous acetonitrile at 298 K (black line) and the fit (red line) using pseudo-first-order kinetic model. *k*_2_ = 161.93 M^−1^S^−1^. Figure S5: Decay of the 505 nm absorbance of TA^+^ClO_4_^−^ (1.0 mM) following the addition of 1c (20 mM) in deaerated anhydrous acetonitrile at 298 K (black line) and the fit (red line) using pseudo-first-order kinetic model. *k*_2_ = 290.83 M^−1^S^−1^; Table S1: All kinetic data for hydride transfer reactions. Scheme S1: ^1^H-NMR and ^13^C-NMR spectra of benzopyran compound 1a (R = H). Scheme S2: ^1^H-NMR and ^13^C-NMR spectra of benzopyran compound 1d (R = Cl). Scheme S3: ^1^H-NMR and ^13^C-NMR spectra of benzopyran compound 1c (R = Me). Scheme S4: ^1^H-NMR and ^13^C-NMR spectra of benzopyran compound 2a. Scheme S5: ^1^H-NMR and ^13^C-NMR spectra of benzopyran compound 3a. Scheme S6: ^1^H-NMR and ^13^C-NMR spectra of benzopyran compound 4a. Scheme S7: Electrochemical spectra of 4 representative compounds. Scheme S8. ITC spectra of 4 representative reactions. Scheme S9: Thermodynamic analysis platform of hydride transfer on the mechanism for 2a. Scheme S10: Thermodynamic analysis platform of hydride transfer on the mechanism for 3a. Scheme S11: Thermodynamic analysis platform of hydride transfer on the mechanism for 4a. References [25,26,27,28] are cited in the Supplementary Materials.
