# Supplementary material for: Essential Rule Derived from Thermodynamics and Kinetics Studies of Benzopyran Compounds
Source: Molecules. 2023 Dec 11;28(24):8039. doi: 10.3390/molecules28248039 (PMC10745646; doi:10.3390/molecules28248039)
Supplement: Supplementary file 1 [file molecules-28-08039-s001.zip › molecules-2684424-supplementary.pdf]

Supporting Information for

Essential rule derived from thermodynamic and kinetic studies of benzopyran compounds

Bao-Long Chen, Xin Hu, and Xiao-Qing Zhu
The State Key Laboratory of Elemento-Organic Chemistry, Collaborative Innovation Center of Chemical Science and Engineering, College of Chemistry, Nankai University, Tianjin 300071, P. R. China.
E-mail: cblnku@163.com; xqzhu@nankai.edu.cn

Table of Contents

**SI.**	**Materials.**	**S2**
**SII.**	**Measurment of Redox Potentials.**	**S2**
**SIII.**	**Isothermal Titration Calorimetry (ITC).**	**S2**
**SIV.**	**^{1}H-NMR and ^{13}C-NMR spectra of representative compounds.**	**S3-S8**
**SV.**	**Electrochemical spectra of representative compounds.**	**S9**
**SVI.**	**ITC spectra of representative reactions.**	**S10-S11**
**SVII.**	**Thermodynamic analysis platform.**	**S11-S12**
**SVIII.**	**Absorbance decays for hydride transfer reactions**	**S13-S15**

SI.Materials.

Solvents and reagents were obtained from commercial sources and used as received unless otherwise noted. Reagent grade acetonitrile was refluxed over $KMnO_4$ and K_2CO_3 for at least eight hours and doubly distilled over P_2O_5 under argon and stored in the glove-box before use. All compounds were synthesized (Figure S1) according to literature methods[1-5]. These references are shown in the original article.

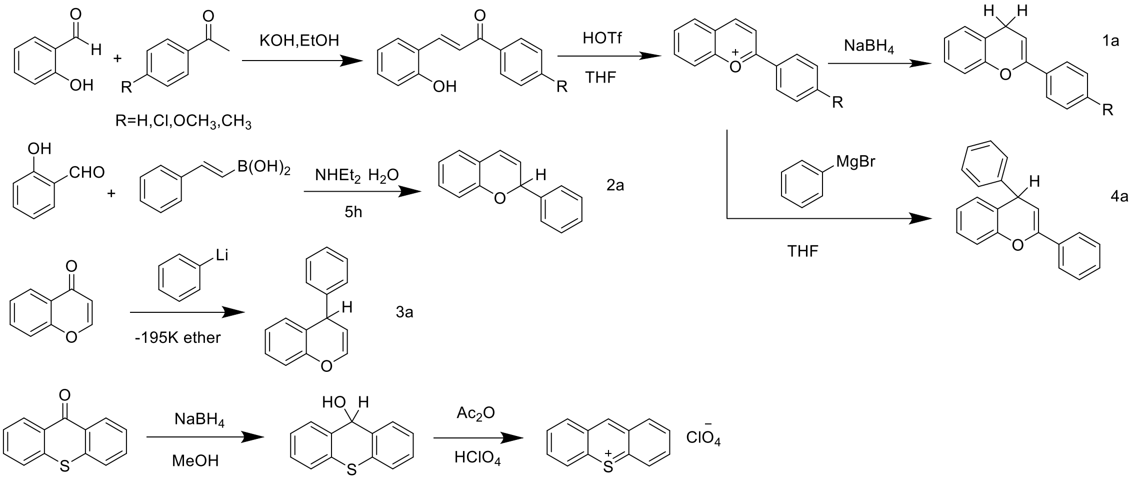

Figure S1. The route of all organic compounds synthesis.

SII.Measurment of Redox Potentials.

The electrochemical experiments were carried out by cyclic voltammetry (CV) and osteryoung square wave voltammetry (OSWV) using BAS100B electrochemical apparatus in deaered acetonitrile under argon atmosphere at 298 K as described previously nBu_4NPF_6 (0.1M) in acetonitrile was employed as the supporting electrolyte. A standard three-electrode cell consists of a glassy carbon disk as work electrode, a platinum wire as a counter electrode, and 0.1 M $AgNO_3$/Ag (in 0.1 M n-Bu_4NPF^{6-} acetonitrile) as reference electrode. The ferrocenium/ferrocene redox couple ($Fc^{+/0}$) was taken as the internal standard. The reproducibilities of the potentials were usually ≤5 mV for ionic species and ≤10 mV for neutral species.

SIII.Isothermal Titration Calorimetry (ITC).

The titration experiments were performed on a CSC4200 isothermal titration calorimeter in acetonitrile at 298 K as described previously The performance of the calorimeter was checked by measuring the standard heat of neutralization of an aqueous solution of sodium hydroxide with a standard aqueous HCl solution，the solvents used in the experiment were anhydrous and anaerobic acetonitrile. $TA^+ClO_4^-$ (1 mM) was used as the titration solution, 1 mL carbonyl compound anion XH was used as the reaction solution. The experiment was determined at 298K, the balance time is 400 s and the titration time is 400 s. The heat of reaction obtained by integrating the area of each peak (except the first peak) in the titration curve. The test was repeated at least 5 times for each sample, and the reaction heat error was ≤±0.5 kcal/mol. Note: typically the first injection shows less heat than expected. This is often due to diffusion across the tip of the needle or to difficulties in positioning the buret drive.

SIV. ^{1}H-NMR and ^{13}C-NMR spectra of representative compounds.

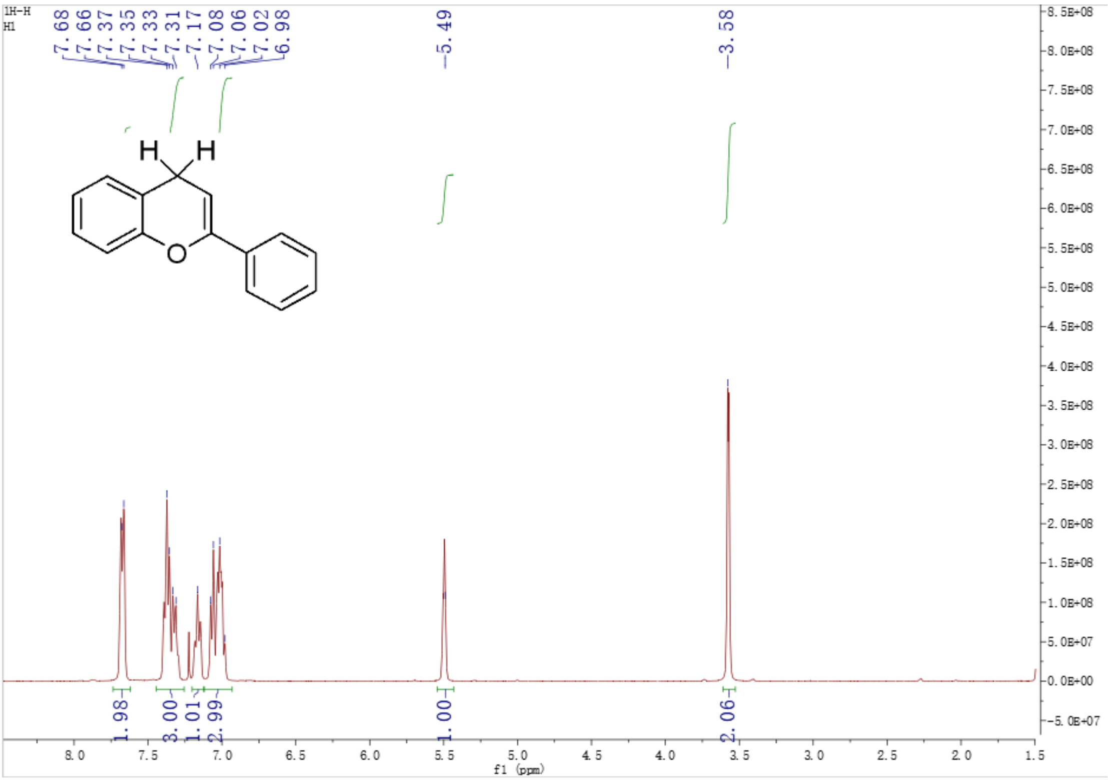

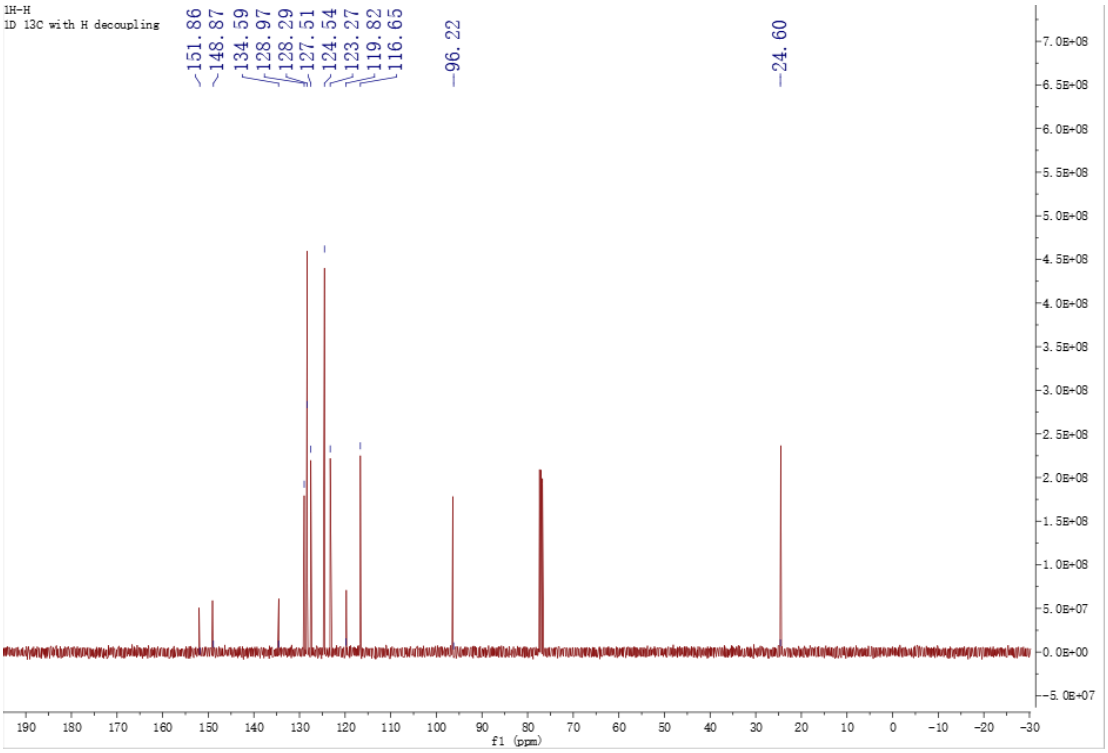

Scheme S1. ^{1}H-NMR and ^{13}C-NMR spectra of benzopyran compound 1a（R=H）.

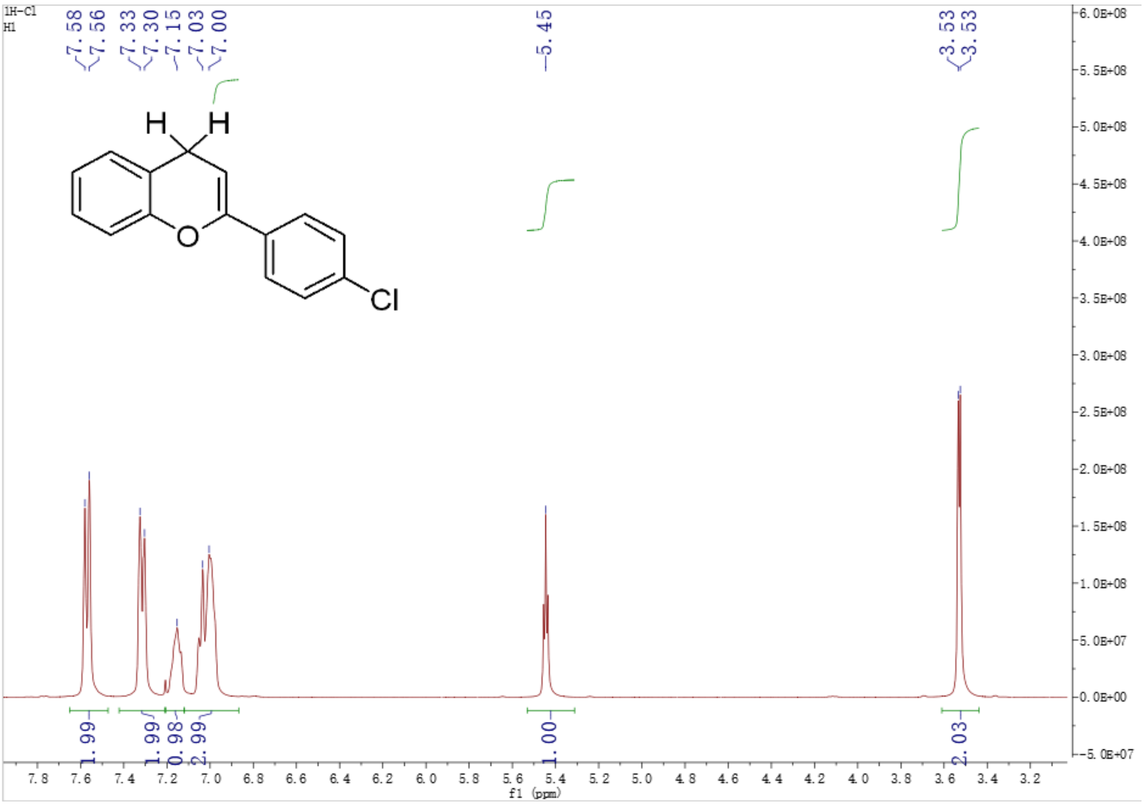

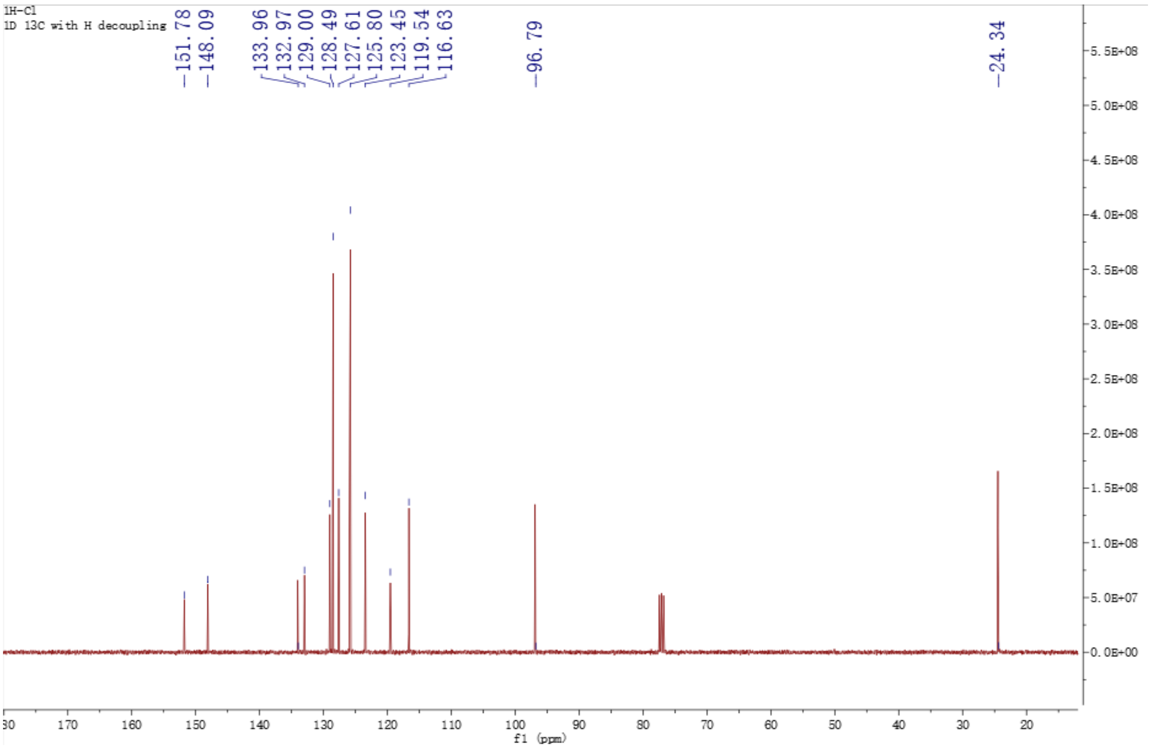

Scheme S2. ^{1}H-NMR and ^{13}C-NMR spectra of benzopyran compound 1d（R=Cl）.

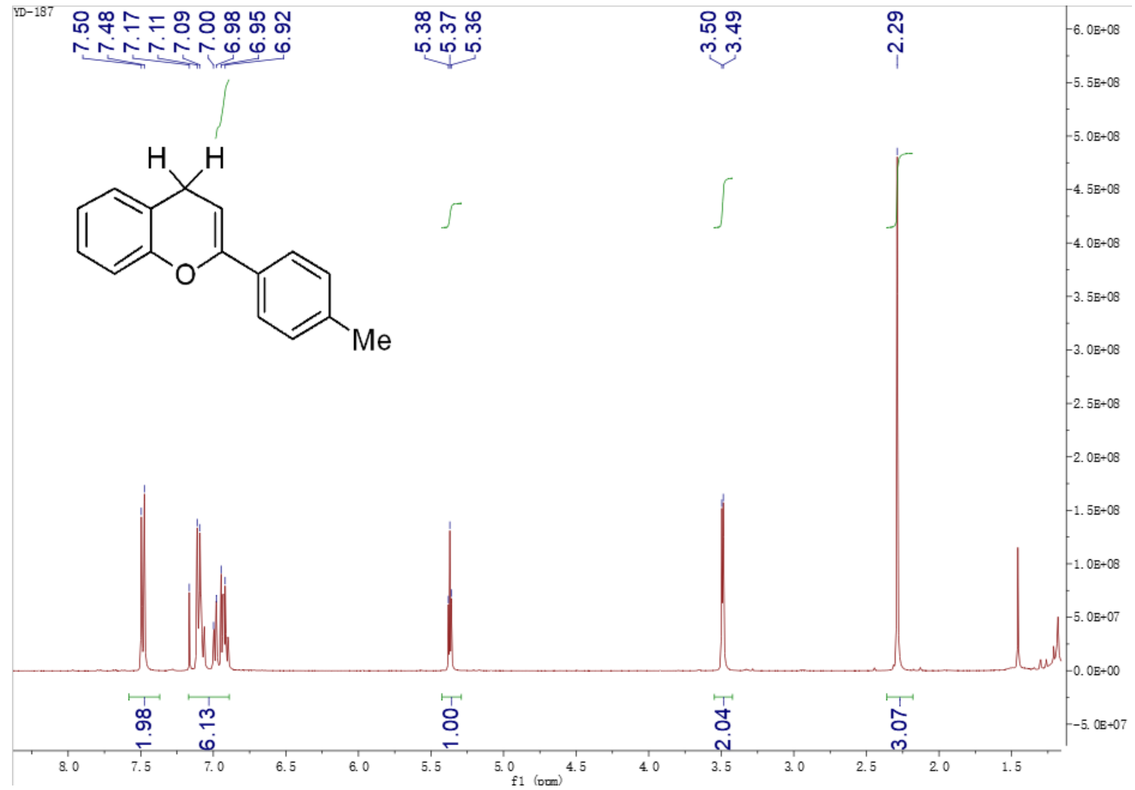

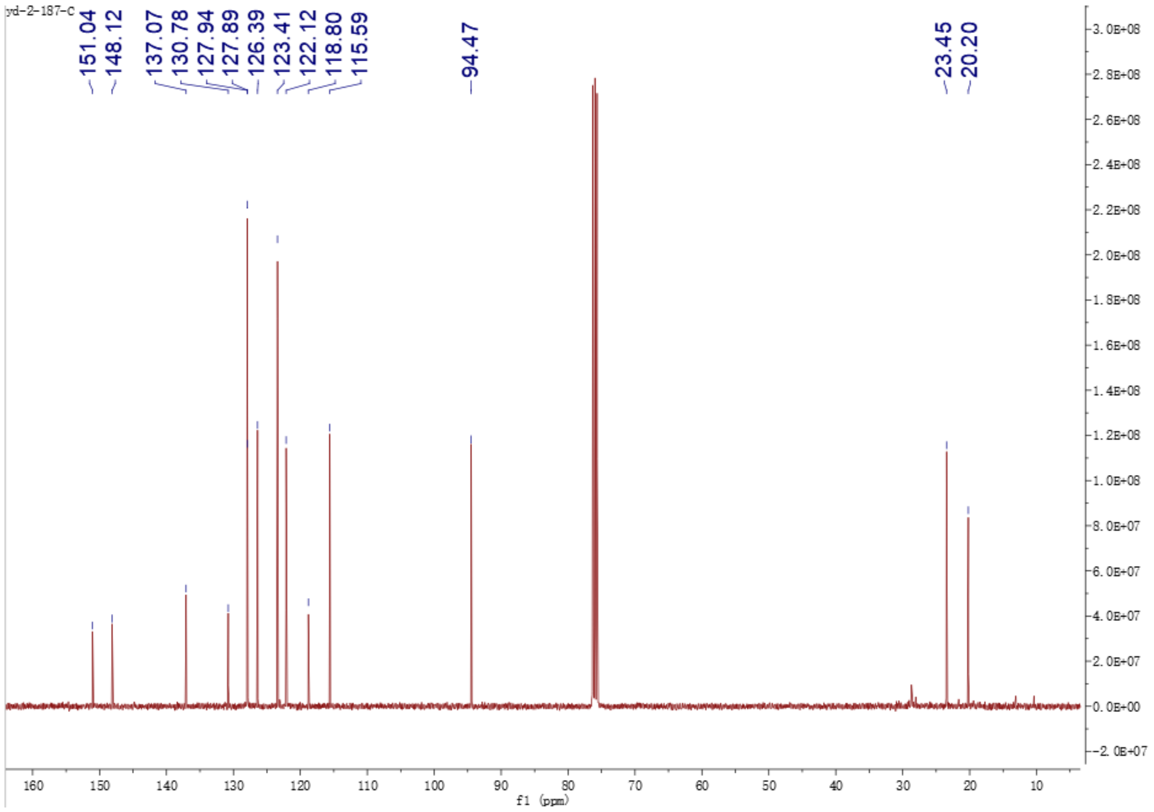

Scheme S3. ^{1}H-NMR and ^{13}C-NMR spectra of benzopyran compound 1c（R=Me）.

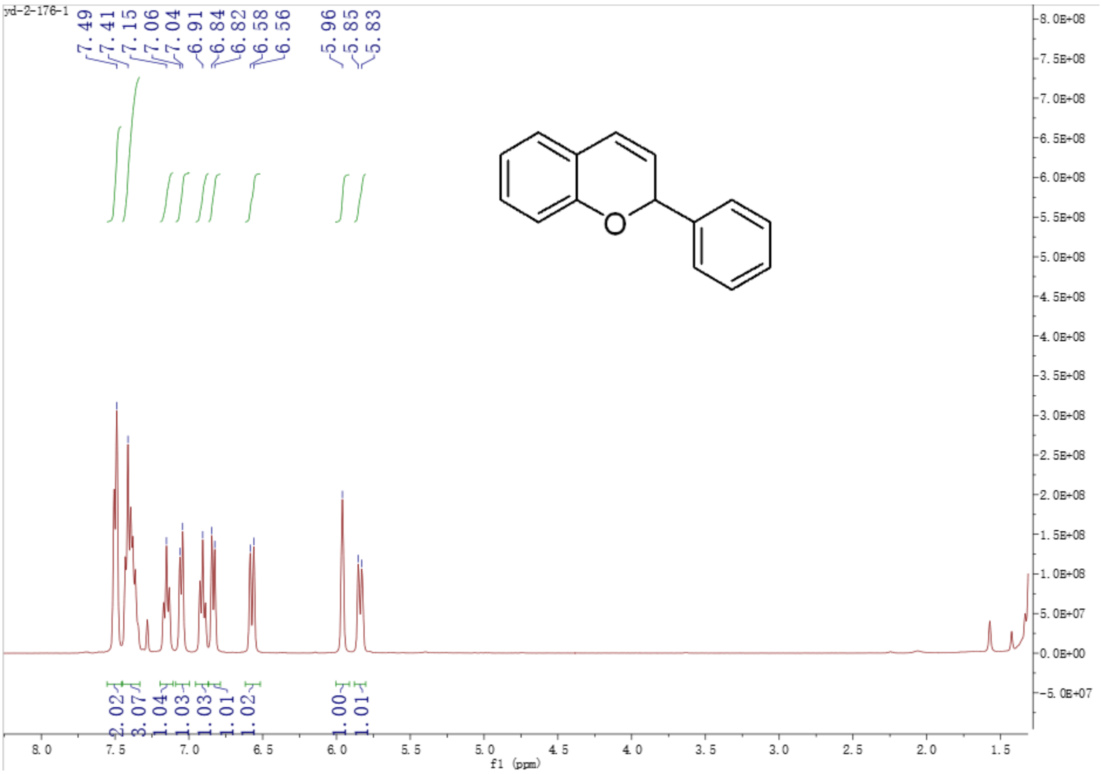

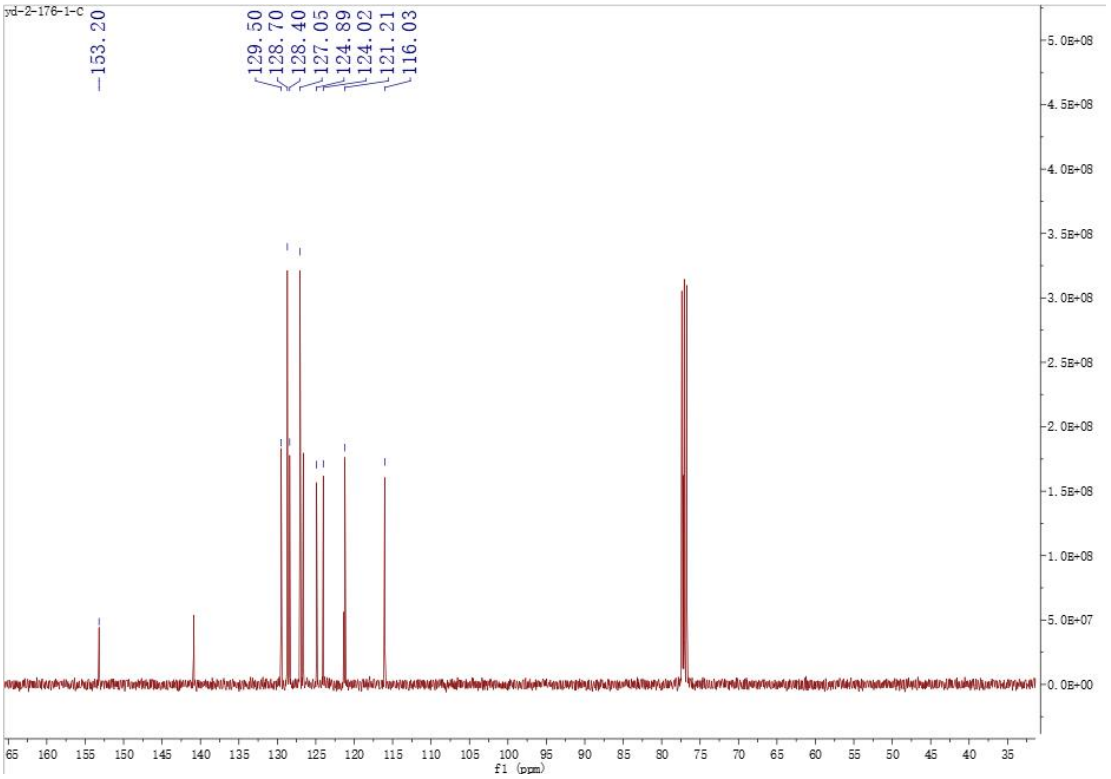

Scheme S4. ^{1}H-NMR and ^{13}C-NMR spectra of benzopyran compound 2a.

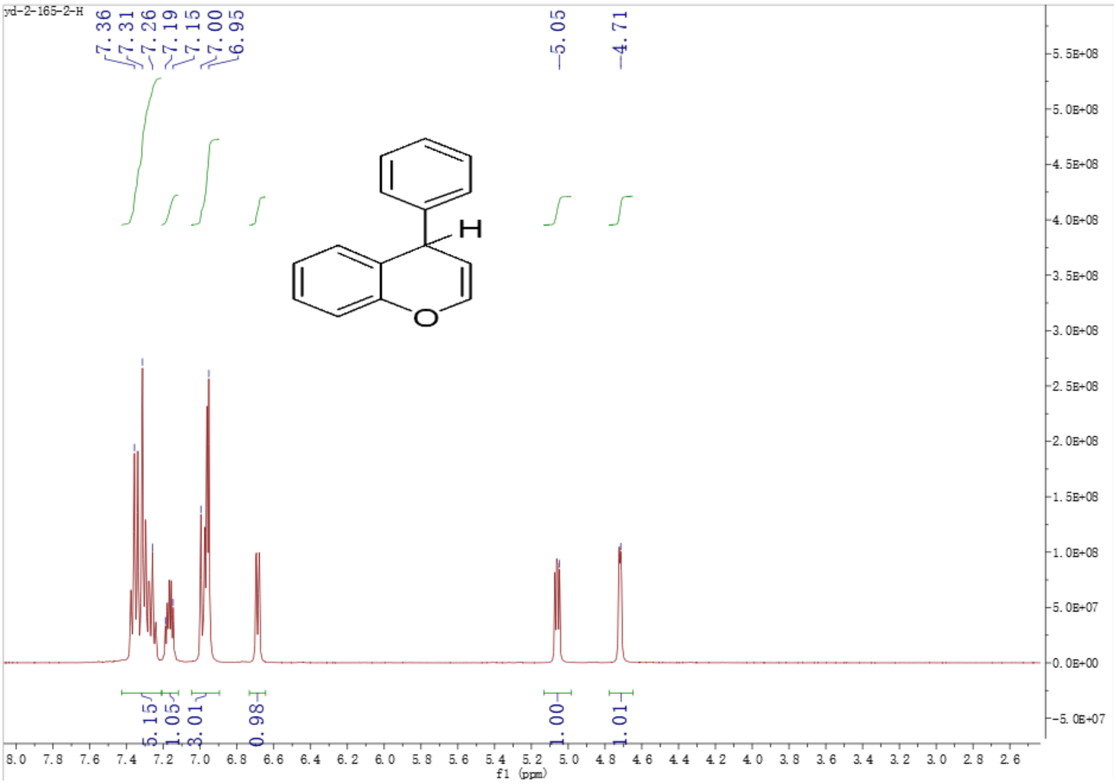

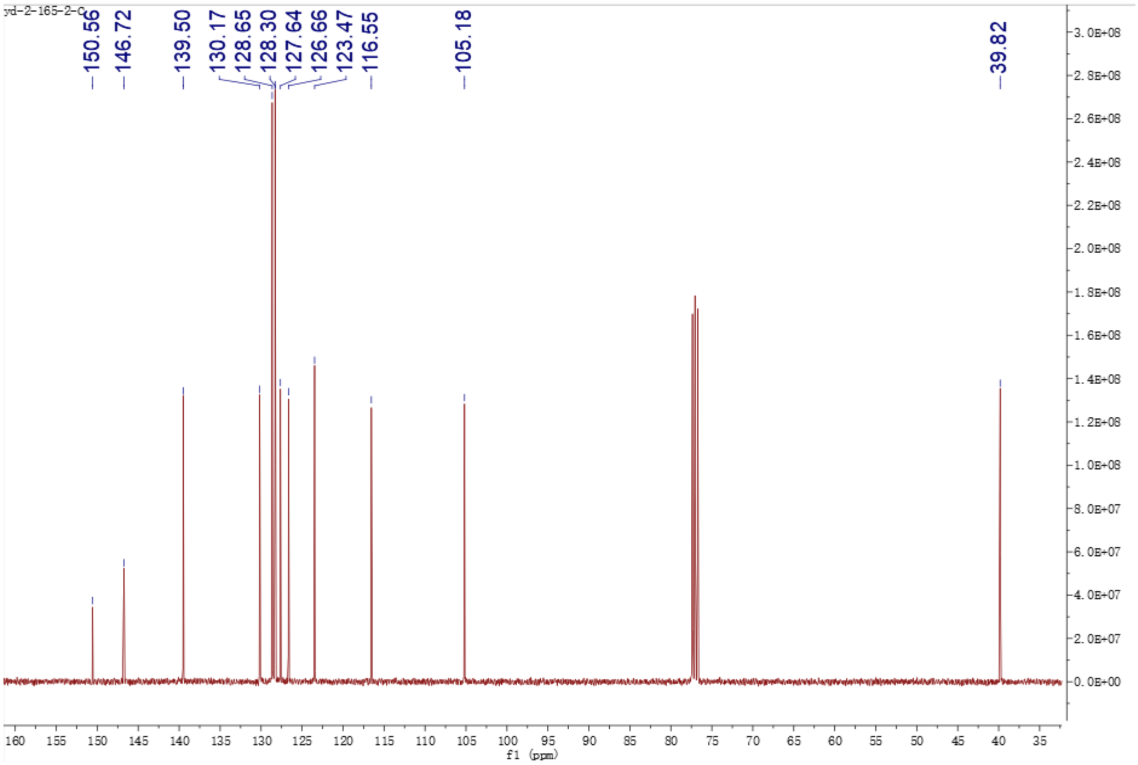

Scheme S5. ^{1}H-NMR and ^{13}C-NMR spectra of benzopyran compound 3a.

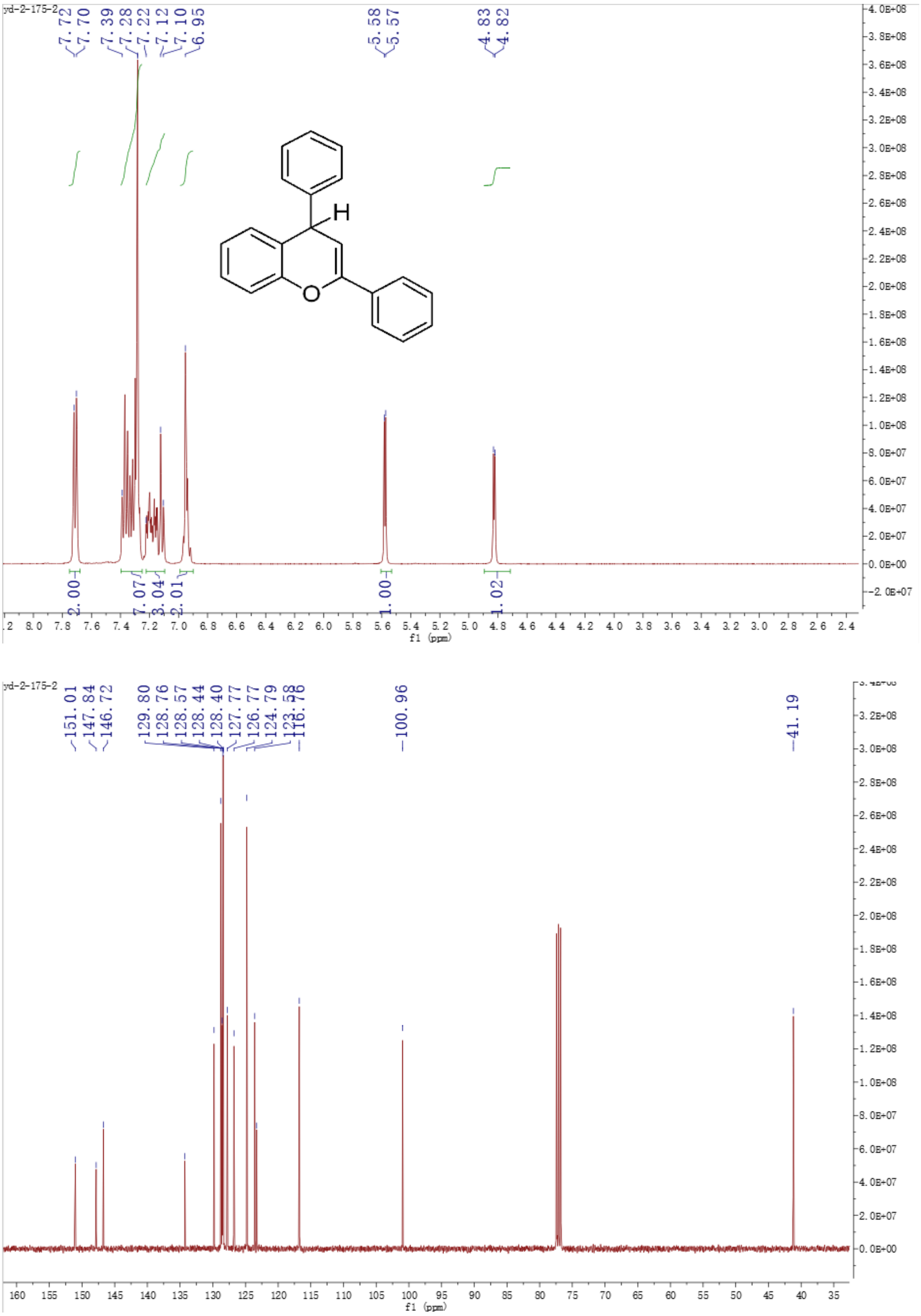

Scheme S6. ^{1}H-NMR and ^{13}C-NMR spectra of benzopyran compound 4a.

SV. Electrochemical spectra of representative compounds.

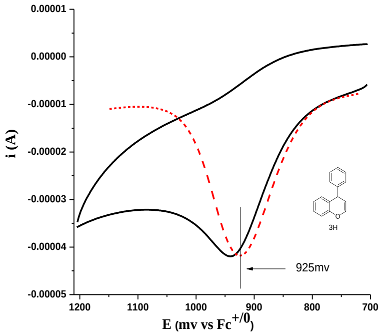

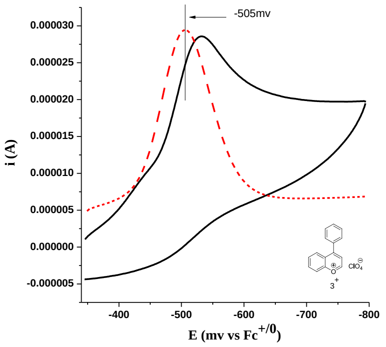

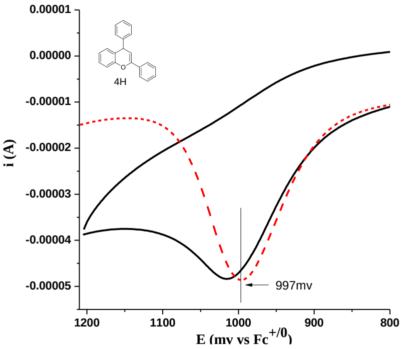

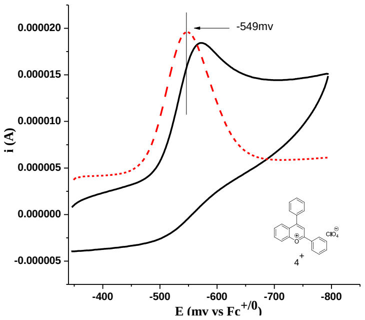

Scheme S7. Electrochemical spectra of 4 representative compounds.

SVI. ITC spectra of representative reactions.

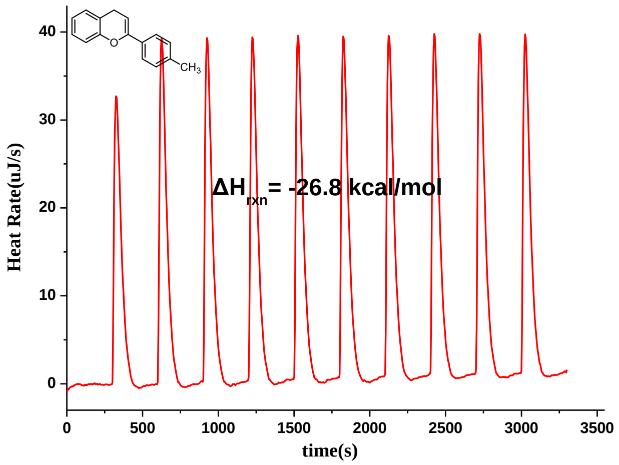

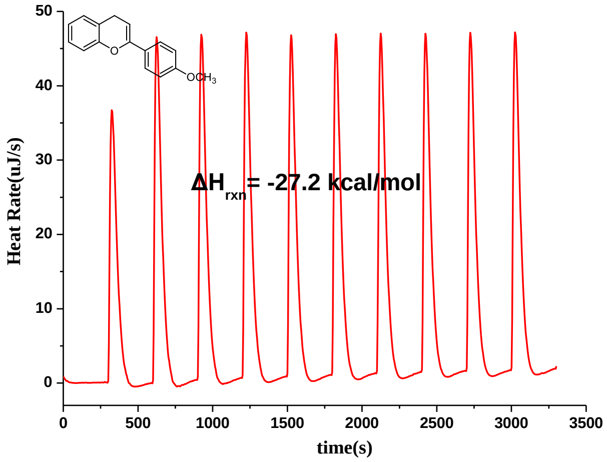

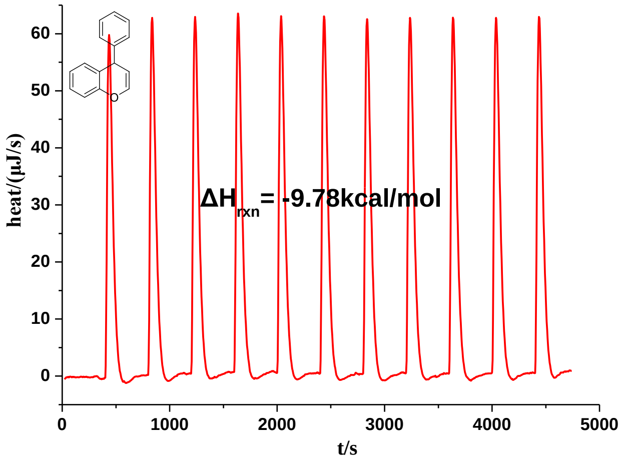

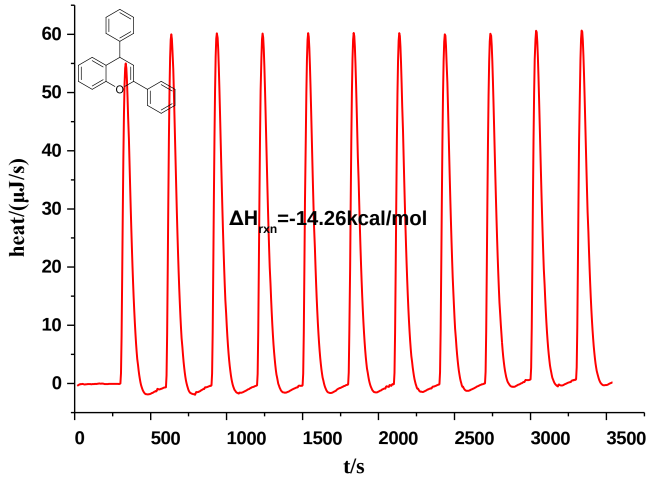

Scheme S8. ITC spectra of 4 representative reactions

SVII. Thermodynamic analysis platform.

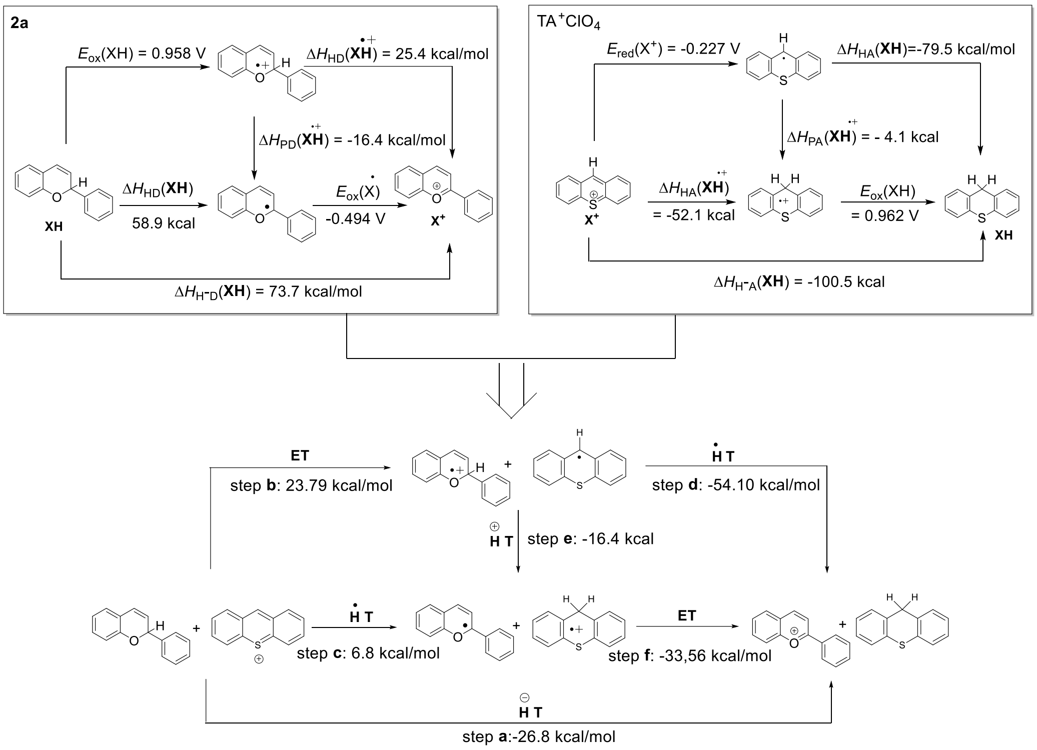

Scheme S9. Thermodynamic analysis platform of hydride transfer on the mechanism for **2a.**

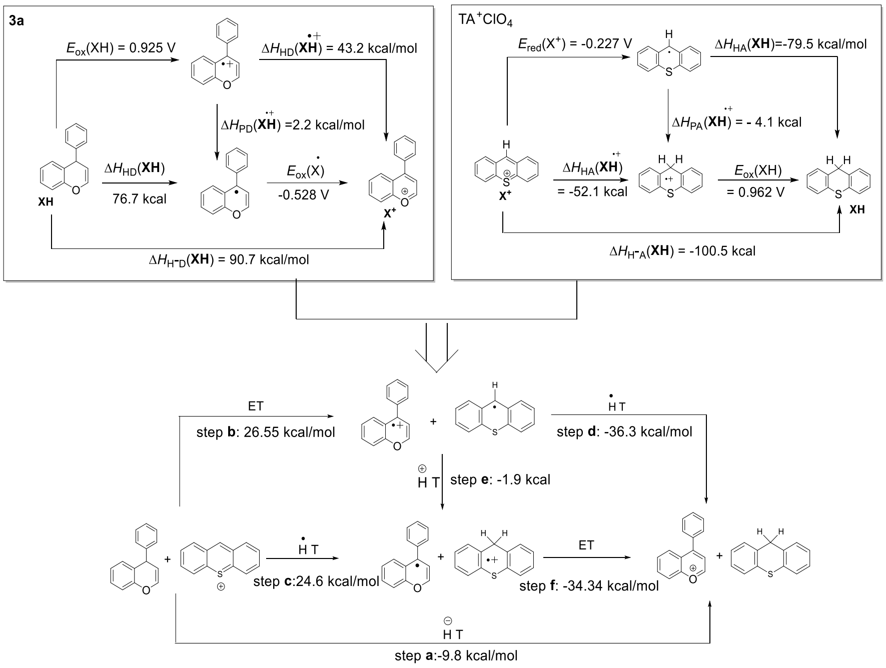

Scheme S10. Thermodynamic analysis platform of hydride transfer on the mechanism for **3a.**

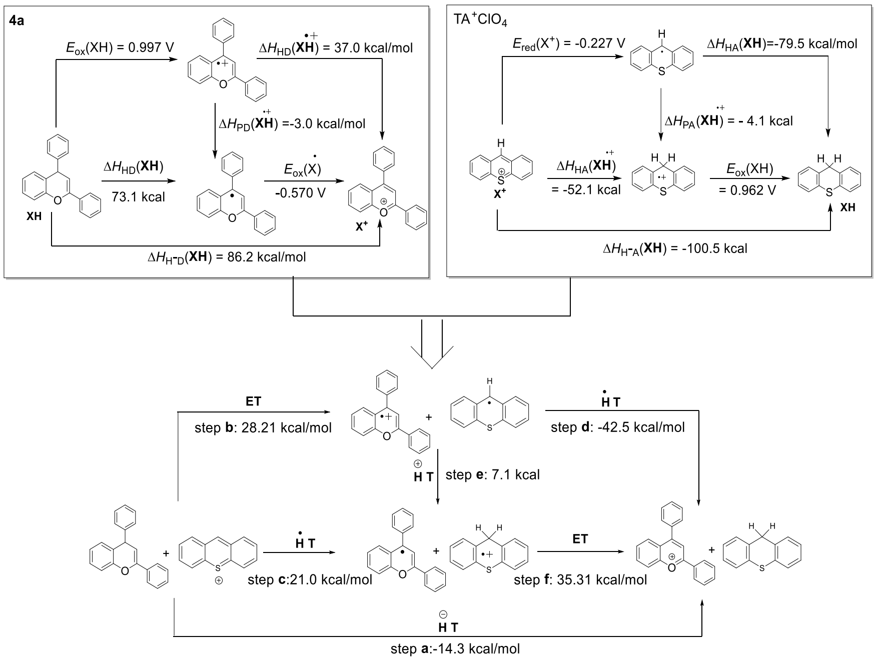

Scheme S11. Thermodynamic analysis platform of hydride transfer on the mechanism for **4a**.

SVIII. Absorbance decays for hydride transfer reactions 1a and $TA^+ClO_4^-$ in acetonitrile at 298 K.

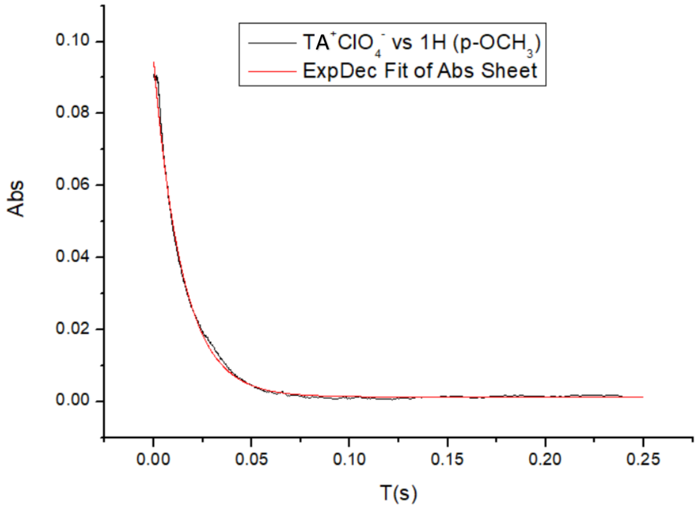

Figure S2. Decay of the 505 nm absorbance of $TA^+ClO_4^-$ (1.0 mM) following the addition of 1b (20 mM) in deaerated anhydrous acetonitrile at 298 K (black line) and the fit (red line) using pseudo-first-order kinetic model. k_2=306.52 $M^{-1}S^{-1}$.

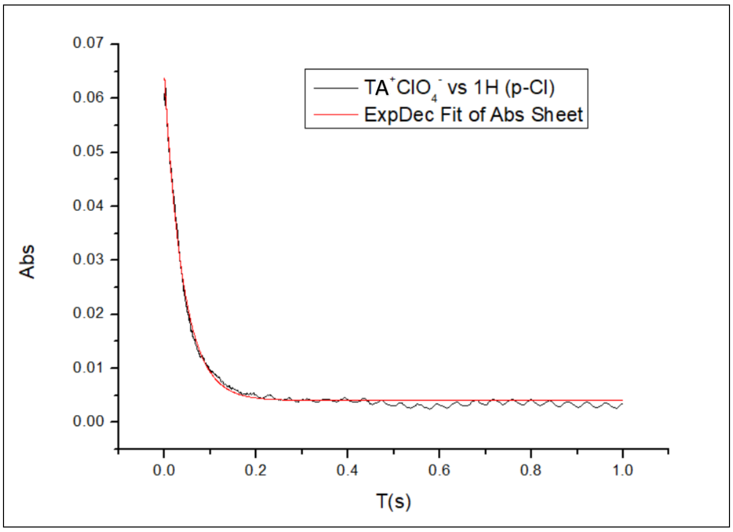

Figure S3. Decay of the 505 nm absorbance of $TA^+ClO_4^-$ (1.0 mM) following the addition of 1d (20 mM) in deaerated anhydrous acetonitrile at 298 K (black line) and the fit (red line) using pseudo-first-order kinetic model. k_2=90.44 $M^{-1}S^{-1}$.

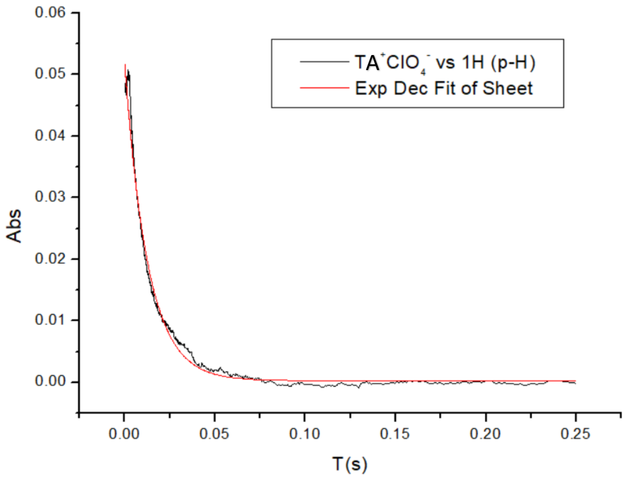

Figure S4. Decay of the 505 nm absorbance of $TA^+ClO_4^-$ (1.0 mM) following the addition of 1a (20 mM) in deaerated anhydrous acetonitrile at 298 K (black line) and the fit (red line) using pseudo-first-order kinetic model. k_2=161.93 $M^{-1}S^{-1}$.

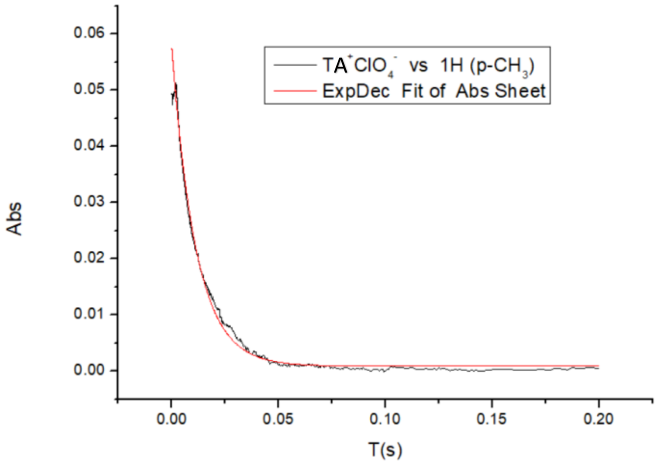

Figure S5. Decay of the 505 nm absorbance of $TA^+ClO_4^-$ (1.0 mM) following the addition of 1c (20 mM) in deaerated anhydrous acetonitrile at 298 K (black line) and the fit (red line) using pseudo-first-order kinetic model. k_2=290.83 $M^{-1}S^{-1}$.

The kinetic runs were performed on an Applied Photophysics SX.18MV-R stopped-flow spectrophotometer at 298 K in acetonitrile. The SX.18MV-R, connected to a superthermostat circulating bath to regulate the temperature of cell compartments, has a dead time of about 1 ms with very high sensitivity. All solutions used for kinetics were prepared in an Ar-filled glovebox. These measurements of the reactions were under pseudo-first-order conditions with the concentrations of excess hydride donor at least 15 times larger than hydride acceptor. The progress of the reaction was monitored by following the decrease of the TA^+absorption at 505nm in the visible region. All runs were repeated more than five times to ensure the reliability of the data.

k_2 (M^{-1} s^{-1}) is the second rate constant of the hydride transfer in acetonitrile at 298 K, derived from experimental measurements using stoped-flow UV−vis spectrometer and the uncertainty is smaller than 5%. Usually, the concentration of hydride acceptor is 0.1mmol/L, the concentration of hydride donor is 2.0mmol/L.

According to Eyring equation:

$k_2 = (k_BT/h)\exp(-\Delta G^{\neq}/RT)$ (1)

When T = 298 K, we substitute the constants into the Eq 1 to obtain the relationship between the activation free energy $\Delta G^{\neq}$ and the second-order rate constant k_2.

$\Delta G^{\neq} = 1.36373\times(12.7926 - \lg k_2)$ (2)

When $k_2 = 161.93\ M^{-1}s^{-1}$, $\Delta G^{\neq} = 14.43$ kcal/mol. All kinetic data are listed in Table S1.

**Compounds**	k_2 **($M^{-1}S^{-1}$)**	$\Delta G^{\neq}$ **(kcal/mol)**
1b	306.52	14.05
1c	290.83	14.09
1a	161.93	14.43
1d	90.44	14.78

Table S1. All kinetic data for hydride transfer reactions.

References

[1] Ehsan, U.M.; Amina, S.; Bilal, A. K.; Muhammad, N. Z.; Ishtiaq, A.; Muhammad, Z. Synthesis, Molecular Docking Studies and Biological Evaluation of 3-Iminoaurones as Acetylcholinesterase and Butyrylcholinesterase Inhibitors. *Lett. Drug. Des. Discov.* **2017**, 14, 1035-1041.

[2] Claudia, F.; Grigoriy, R.; Herbert, M. Kinetics of the Reactions of Flavylium Ions with π-Nucleophiles. *Eur. J. Org. Chem.* **2001**, 4451-4456.

[3] Nuno, R. C.; Pedro, M. C.; Vania, A.; Teresa, D.; Luis, F. V.; Pedro M.P. Water as the reaction medium for multicomponent reactions basedon boronic acids. *Tetrahedron*. **2010**,66, 2736–2745.

[4] Stan, S. H.; Sami, E. F. Tandem Arylation-Reduction of Acyl Heterocycles Convenient Synthesis of Benzyl [1,2]. *J. Heterocyclic. Chem.* **1987**, 24, 1205-1213.

[5] Wu, Y.-C.; Liu, L.; Liu,Y.-L.; Wang, D.; Chen, Y.-J. TFA-Mediated Tandem Friedel-Crafts Alkylation/Cyclization/Hydrogen Transfer Process for the Synthesis of Flavylium Compounds. *J. Org. Chem.* **2007**, 72, 9383-9386 9383
